# Sequential Appearance and Isolation of a SARS-CoV-2 Recombinant between Two Major SARS-CoV-2 Variants in a Chronically Infected Immunocompromised Patient

**DOI:** 10.3390/v14061266

**Published:** 2022-06-10

**Authors:** Emilie Burel, Philippe Colson, Jean-Christophe Lagier, Anthony Levasseur, Marielle Bedotto, Philippe Lavrard-Meyer, Pierre-Edouard Fournier, Bernard La Scola, Didier Raoult

**Affiliations:** 1 IHU Méditerranée Infection, 19-21 Boulevard Jean Moulin, 13005 Marseille, France; burel.emilie@hotmail.fr (E.B.); philippe.colson@univ-amu.fr (P.C.); jean-christophe.lagier@univ-amu.fr (J.-C.L.); anthony.levasseur@univ-amu.fr (A.L.); marielle.bedotto@gmail.com (M.B.); philippe.lavrard-meyer@ap-hm.fr (P.L.-M.); pierre-edouard.fournier@univ-amu.fr (P.-E.F.); 2 Microbes Evolution Phylogeny and Infections (MEPHI), Institut de Recherche Pour le Développement (IRD), Aix-Marseille University, 27 Boulevard Jean Moulin, 13005 Marseille, France; 3 Assistance Publique-Hôpitaux de Marseille (AP-HM), 264 rue Saint-Pierre, 13005 Marseille, France; 4 Vecteurs-Infections Tropicales et Méditerranéennes (VITROME), Institut de Recherche Pour le Développement (IRD), Aix-Marseille University, 27 Boulevard Jean Moulin, 13005 Marseille, France

**Keywords:** SARS-CoV-2, variant, recombination, chronic infection, immunosuppression

## Abstract

Genetic recombination is a major evolutionary mechanism among RNA viruses, and it is common in coronaviruses, including those infecting humans. A few SARS-CoV-2 recombinants have been reported to date whose genome harbored combinations of mutations from different mutants or variants, but only a single patient’s sample was analyzed, and the virus was not isolated. Here, we report the gradual emergence of a hybrid genome of B.1.160 and Alpha variants in a lymphoma patient chronically infected for 14 months, and we isolated the recombinant virus. The hybrid genome was obtained by next-generation sequencing, and the recombination sites were confirmed by PCR. This consisted of a parental B.1.160 backbone interspersed with two fragments, including the spike gene, from an Alpha variant. An analysis of seven sequential samples from the patient decoded the recombination steps, including the initial infection with a B.1.160 variant, then a concurrent infection with this variant and an Alpha variant, the generation of hybrid genomes, and eventually the emergence of a predominant recombinant virus isolated at the end of the patient’s follow-up. This case exemplifies the recombination process of SARS-CoV-2 in real life, and it calls for intensifying the genomic surveillance in patients coinfected with different SARS-CoV-2 variants, and more generally with several RNA viruses, as this may lead to the appearance of new viruses.

## 1. Introduction

A major evolutionary mechanism of RNA viruses is genetic recombination [1,2]. Recombinations are extremely common in coronaviruses and have been implicated in the emergence of several genotypes, including endemic human coronaviruses [3,4,5,6]. The involvement of genetic recombination in the origin of SARS-CoV-2 is also suspected [7]. Regarding SARS-CoV-2, coinfection in the same patient with distinct variants has been reported [8,9,10,11,12,13,14]. In addition, several studies have described or suspected genetic recombinations for this virus [10,13,14,15,16,17,18,19,20,21,22,23,24,25]. However, most of these recombinants have relied solely on the coexistence of signature mutations of different SARS-CoV-2 variants in genomes obtained from a single patient’s sample, and they were not isolated in culture. Since January 2020, our laboratory has screened more than one million respiratory specimens for SARS-CoV-2 infection by real-time reverse transcription-PCR (qPCR) without interruption or limited capacity, including for all patients sampled in our institute and in the Marseille public hospitals [26,27]. This has provided us with sequential samples from multiple patients, and enabled us to detect reinfections, and prolonged or even chronic infections in severely immunocompromised patients [28,29,30]. Here, we report the gradual emergence of a recombinant SARS-CoV-2 involving two variants in a lymphoma patient chronically infected over a period of 14 months, and the isolation of the recombinant virus in culture.

## 2. Results

### 2.1. Chronic SARS-CoV-2-Infection in a Severely Immunocompromised Patient

A 56-year-old immunocompromised male had an uncontrolled SARS-CoV-2 infection for 14 months until death (Supplementary Material: Supplementary Methods and Results). He was diagnosed in 2017 with mixed Hodgkin and follicular lymphoma and was in complete remission of the Hodgkin lymphoma following chemotherapy, and on maintenance therapy. Follicular lymphoma progression led to the administration of lenalidomide plus rituximab in 2021, and pembrolizumab was also administered. Progressive multifocal leukoencephalitis was diagnosed in May 2021. In August 2020, the patient developed severe SARS-CoV-2-associated pneumonia, leading to admission in the intensive care unit. He improved clinically but viral clearance did not occur, and SARS-CoV-2 RNA remained detectable by qPCR on most nasopharyngeal samples collected between September 2020 and December 2021. qPCR was negative in February 2021 but positive when re-tested in April 2021, and then only transiently negative for ≤3 days. COVID-19 patient convalescent plasma was administered during hospitalization outside our institute in October 2020 and April 2021, and hydroxychloroquine was administered in July 2021. The history of SARS-CoV-2 infection did not lead to vaccine administration. The patient died unfortunately in December 2021 from the complications of his hematological and neurological diseases.

### 2.2. Evidence of Hybrids of Variants

**Figure 1:**
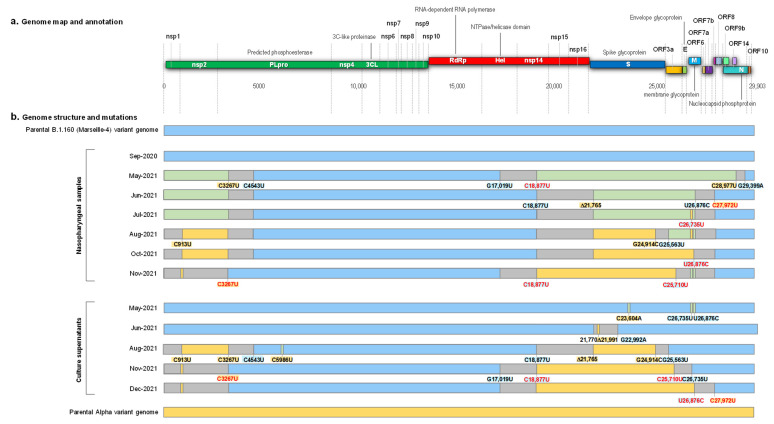
Schematic representation of the structure of the SARS-CoV-2 genomes obtained from the nasopharyngeal samples and from the culture supernatants, as well as of the recombination events over time, in reference to parental genomes of the B.1.160 and Alpha variants. (**a**) Genome map and annotation; (**b**) Genome structure and mutations. Blue color of rectangles indicates sequences from a B.1.160 variant; yellow color indicates sequences from an Alpha variant; green color indicates co-detection of sequences from a B.1.160 variant and from an Alpha variant; grey color indicates sequences from indeterminate origin. Signature mutations from the B.1.160 and Alpha variants are indicated by a blue background and a yellow background, respectively. Signature mutations that are absent are indicated by a red font. Δ21,765: -6 nucleotides; Δ21,991: -3 nucleotides. Nsp, nonstructural protein; ORF, open reading frame.

**Figure 2:**
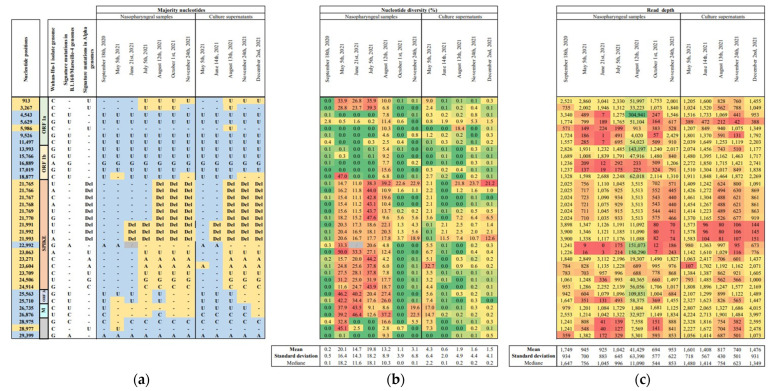
Majority nucleotides (**a**) nucleotide diversity (**b**) and sequencing depth (**c**) for sequences obtained from the respiratory samples and the culture supernatant at nucleotide positions of the SARS-CoV-2 genome that harbor signature mutations of the B.1.160 or Alpha variants. Del, nucleotide deletion. Nucleotide positions are in reference to the genome of the Wuhan-Hu-1 isolate GenBank accession no. NC_045512.2. (**b**) Nucleotide diversity is the proportion of sequence reads that do not harbor the consensus (majority) nucleotide. (**c**) Read depth is the number of reads covering a given nucleotide position.

After 14 months of infection, we identified a virus whose genome was a hybrid of two known variants, B.1.160 (according to PANGOLIN (Phylogenetic Assignment of Named Global Outbreak Lineages) lineage (https://cov-lineages.org/resources/pangolin.html; accessed on 20 October 2021) [31]) (a.k.a. Nextstrain clade (https://nextstrain.org/; accessed on 20 October 2021) [32] 20A.EU2, or Marseille-4 [27]) and Alpha (according to the WHO denomination (https://www.who.int/fr/activities/tracking-SARS-CoV-2-variants; accessed on 20 October 2021) (a.k.a. 20I or B.1.1.7)) (Figure 1 and Figure 2).

This hybrid genome was obtained from the respiratory samples by next-generation sequencing, as previously described [27]. In addition, the hybrid virus was isolated in culture, as previously described [33]. The hybrid genome sequence consisted of a B.1.160 variant matrix, of which two regions, the first one being located at the 5′ tip of the genome and containing the synonymous mutation C913U, and the second one spanning from positions 17,109–18,877 to positions 25,710–27,972, were replaced by those of an Alpha variant (Figure 1 and Figure 2; Supplementary Material: Figures S1 and S2). All eight signature mutations of the Alpha variant were detected in the spike gene in the absence of the S477N mutation that is a signature of the B.1.160 variant. Nucleotide diversity at the 35 positions harboring signature mutations of the Alpha or B.1.160 variants was low (mean (±standard deviation) value of 3.1 ± 6.8%) (Figure 2; Supplementary Material: Figures S1 and S2), indicating that the hybrid content of the genome was not explained by a co-infection from the two variants or by contamination. These findings indicated that this mosaic genome was the result of recombinations between parental genomes of the B.1.160 and Alpha variants.

**Figure 3:**
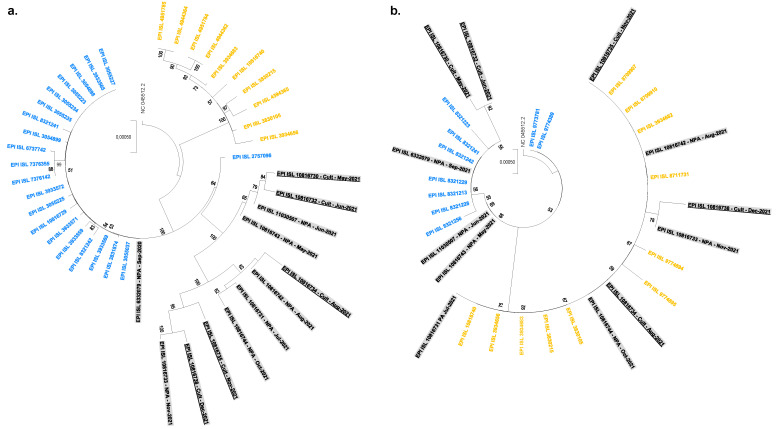
Phylogenetic analyses based on SARS-CoV-2 genomes (**a**) and spike gene sequences (**b**). Sequences obtained from the case-patient are indicated by a grey background, and those obtained from cultures are underlined. Other sequences from our SARS-CoV-2 sequence database are indicated by a blue font when classified as of the B.1.160 variant, and by a yellow font when classified as of the Alpha variant. Sequences are labeled with their GISAID (https://www.gisaid.org/; accessed on 18 May 2022) [34] identifiers. Trees are rooted with the genome of the Wuhan-Hu-1 isolate GenBank accession no. NC_045512.2.2.3. Genome of the initial virus.

By analyzing the sequential samples available from this patient, we were able to determine that he was first infected with the B.1.160 variant, which was epidemic at the time of diagnosis of his infection in September 2020. This variant predominated in our region from August 2020 until January 2021 and was replaced by the Alpha variant, which emerged in December 2020 [27]. SARS-CoV-2 could not be isolated retrospectively from this sample, but its genome was typical of a B.1.160 variant and displayed no significant nucleotide diversity (mean, 0.2 ± 0.5%). It was classified by phylogeny as a B.1.160 variant (Figure 3).

Unfortunately, although SARS-CoV-2 qPCR was still positive in another laboratory in January 2021, the sample was unavailable. Therefore, we were not able to confirm whether Marseille-4 and Alpha variants were co-infecting the patient at this time nor the duration of co-infection, and we did not obtain the genome nor an isolate of the Alpha variant. The closest sample in time to the initial one dated from May 2021, 8 months after the first SARS-CoV-2 diagnosis, and it already demonstrated a mosaicism between genomes of the B.1.160 and Alpha variants (Figure 1 and Figure 2).

### 2.3. Steps in Generation of the Recombinant

We used three procedures to characterize the different recombination steps by analyzing seven sequential respiratory samples collected from the patient (Table 1 and Table 2). First, through sequencing from the respiratory samples of the viral genomes; second, sequencing from the respiratory samples of PCR products overlapping the putative recombination sites; third, a viral culture with sequencing of the genomes of the isolates. These approaches allowed us to evidence that several viruses and recombinant forms had coexisted in the sequential samples, as signature mutations of the two variants were co-detected at multiple positions, with a nucleotide diversity that reached high levels and that evolved over time (Figure 2; Supplementary Material: Supplementary Results and Figure S1). We observed an evolution towards the genome sequence of the recombinant virus that predominated at the end of the patient’s follow-up, following recombination events at three sites between parental genomes of B.1.160 and Alpha variants, with a low level of nucleotide diversity observed at that time at the positions harboring signature mutations of these variants. 

To further support the breakpoints identified between the parental genomes of the Alpha and Marseille-4 variants, we generated Marseille-4 variant/Alpha variant chimeric sequences of the regions overlapping the three putative recombination sites by PCR amplification (Supplementary Material: Figures S3–S5), followed by next-generation sequencing with Nanopore Technology on a gridION instrument, as previously described [27]. For the amplicon corresponding to positions 3100–4570 of the genome, 56 and 87% of the reads were chimeras harboring both Alpha (C3267U) and Marseille-4 (C4543U) signature mutations from the respiratory samples collected in 5 May and in 12 August 2021, respectively (Supplementary Material: Figures S3 and S4). For the amplicon corresponding to positions 24,880–29,010, 70% of the reads were chimeras harboring the Alpha mutation G24914C and the Marseille-4 mutations G25563U, C25710U, C26735U, U26876C and G28975C, and 24% were chimeras harboring the Alpha mutation G24914C and the Marseille-4 mutation G28975C (Supplementary Material: Figure S5). No PCR amplification was obtained for the region between positions 21,417–23,901. Next-generation sequencing was also carried out using a metagenomic approach with the Nanopore technology to obtain large reads and detect additional Marseille-4 variant/Alpha variant chimeras. For the first previously identified recombination region, four reads with a length ranging between 1855 and 11,979 nucleotides were obtained that harbored signature mutations of the Alpha (C3267U) and Marseille-4 (C4543U) variants. For the third recombination region, four reads with a length between 1486–8143 nucleotides were obtained that harbored signature mutations of the Alpha (G24914C) and Marseille-4 (G25563U) variants (available from URL: https://www.mediterranee-infection.com/sars-cov-2-recombinant/; accessed on 18 May 2022). No reads were obtained that covered the Marseille-4 mutation C18877U and the Alpha deletion UACAUG21765. Therefore, several breakpoints between the parental genomes of Alpha and Marseille-4 variants were supported by the presence of chimeric reads.

## 3. Discussion

We highlight here, in an immunocompromised lymphoma patient chronically infected with SARS-CoV-2 and who received several treatments, the presence of a virus hybrid of two known variants, B.1.160 and Alpha, which successively predominated in our region during the follow-up period of this patient [27,35]. The absence of available samples covering the period between the diagnosis of infection by the B.1.160 variant and first evidence of a hybrid genome did not allow us to date the superinfection by the Alpha variant. The signature mutations of the Alpha variant observed in the hybrid genomes between 8 and 14 months cannot have occurred randomly considering their number, distribution along the genome, and their majority presence, and their location indicates recombinations in three regions. In addition, genomic analyses carried out for sequential respiratory samples and viral cultures demonstrate the successive presence of several viruses with hybrid genomes, one of them having established itself in this patient and the one that continued circulating until his death.

We believe that this observation, which sheds light on the recombination mechanism of RNA viruses, is significant, and to our knowledge, this is the first study describing, through the analysis of sequential samples over more than a year, the generation of recombinant SARS-CoV-2 and its isolation in culture. Sixteen interlineage recombinants between the Alpha variant and non-Alpha viruses were reported in 2021 in the UK, of 279,000 genomes analyzed [8]. In addition, 1175 (0.2%) putative recombinant genomes were identified among 537,360 genomes, and it was reported that up to 5% of SARS-CoV-2 that circulated in the USA and UK might be recombinants [18]. Moreover, the number of cases that capture detection of recombinant genomes is growing [10,13,14,15,16,17,18,19,20,21,22,23,24,25], including with recombinant events involving or between Omicron variants [36,37,38,39], which highlights the importance of recombination in the evolution of SARS-CoV-2. Besides recombination between SARS-CoV-2 infecting the same human cells, other evolutionary pathways may exist [39]. For instance, different evolutionary trajectories in distinct cell types of the same infected host have been reported [40], and coronaviruses have been reported to harbor a sequence shared with four different families of positive-sense single-stranded RNA viruses and that is putatively shared with insects [41].

Such natural mosaicisms in these viruses make it possible to understand the emergence of RNA viruses and should lead to a strengthening of genomic surveillance in patients, especially in immunocompromised long-term viral carriers, presenting with coinfections by several RNA viruses, as observed in patients infected with several respiratory viruses including SARS-CoV-2, endemic human coronaviruses, influenza viruses, or rhinoviruses [42]. Such infectious episodes could perhaps lead to the emergence of new emerging viruses, as has been, for instance, reported for enteroviruses of humans and great apes [43]. 

## 4. Materials and Methods

### 4.1. SARS-CoV-2 Genome Sequencing

SARS-CoV-2 genome sequencing was performed as previously described. Briefly, viral RNA was extracted from 200 μL of nasopharyngeal swab fluid using the EZ1 Virus Mini kit v2.0 on an EZ1 Advanced XL instrument (Qiagen, Courtaboeuf, France) or using the MagMax Viral/Pathogen Nucleic Acid Isolation kit on the KingFisher Flex system (Thermo Fisher Scientific, Waltham, MA, USA), following the manufacturer’s instructions. SARS-CoV-2 genome sequences were obtained by next-generation sequencing with various procedures with the Illumina COVIDSeq protocol on a NovaSeq 6000 instrument (Illumina Inc., San Diego, CA, USA), or by multiplex PCR with ARTIC nCoV-2019 V3 Panel primers (IDT, Coralville, IA, USA) were combined with the Oxford Nanopore technology (ONT) on a GridION instrument (Oxford Nanopore Technologies Ltd., Oxford, UK), as previously described [14,27]. After its extraction, viral RNA was reverse-transcribed according to the COVIDSeq protocol (Illumina Inc., San Diego, CA, USA) or by using the LunaScript RT SuperMix kit (New England Biolabs, Ipswich, MA, USA) when performing next-generation sequencing with the Nanopore technology, following the manufacturer’s recommendations.

### 4.2. Genome Analysis

After using the Nanopore technology combined with the ARTIC protocol, fastq files were processed using the ARTIC field bioinformatics pipeline (ARTIC-nCoV-bioinformaticsSOP-v1.1.0, Nick Loman, Will Rowe, Andrew Rambaut, the ZiBRA Project and the ARTIC project, University of Birmingham, UK, https://artic.network/ncov-2019/ncov2019-bioinformatics-sop.html; https://github.com/artic-network/fieldbioinformatics; accessed on 18 May 2022), as previously described [14,27]. Next-generation sequencing reads were base-called using Guppy (4.0.14) and were aligned to the genome of the Wuhan-Hu-1isolate, GenBank accession No. MN908947.3, using minimap2 (v2.17-r941) (https://github.com/lh3/minimap2; accessed on 18 May 2022, Dana-Farber Cancer Institute of Boston, USA) [44]. The ARTIC tool align_trim was used to softmask primers from read alignment and cap sequencing depth at a maximum of 400-fold coverage. Consensus-level variant candidates were identified using a threshold of 70% and the Medaka (v.0.11.5) workflow, developed by ARTIC (Nick Loman, Will Rowe, Andrew Rambaut, the ZiBRA Project and the ARTIC project, University of Birmingham, UK, https://github.com/artic-network/artic-ncov2019; accessed on 18 May 2022). From the unique sequence obtained with the ARTIC-Nanopore technology, a sorted bam file was loaded on the CLC Genomics workbench v7 software and a tsv file was then exported. NovaSeq reads were basecalled using the Dragen Bcl Convert pipeline (v3.9.3; https://emea.support.illumina.com/sequencing/sequencing_software/bcl-convert.html; accessed on 18 May 2022 (Illumina Inc., San Diego, CA, USA). Mapping was carried out on the Wuhan-Hu-1 isolate genome with the bwa-mem2 tool (v2.2.1; https://github.com/bwa-mem2/bwa-mem2; accessed on 18 May 2022, Heng Li, Harvard, USA) and was cleaned with Samtools (v1.13; https://www.htslib.org/; accessed on 18 May 2022) [45]. Variant calling was performed with freebayes (v1.3.5; https://github.com/freebayes/freebayes; accessed on 18 May 2022, Erik Garrison, Gabor Marth, University of Tennessee and Utah) [46] and consensus genomes were built with Bcftools (v1.13; https://samtools.github.io/bcftools/bcftools.html; accessed on 18 May 2022, 2012–2021 Genome Research Ltd.). Freebayes results were filtered with a threshold of 70% for the majority nucleotide. A tsv file was generated using an in-house Python script. The clade was designated at the consensus level with the Nextclade online tool (https://clades.nextstrain.org/; accessed on 18 May 2022, 2020 Nextstrain developers, Swiss Institute of Bioinformatics) [32,47] and an in-house Python script allowed for the detection of variants and the hybrids of variants. At the sub-consensus level, variant frequencies that were compared to the Wuhan-Hu-1 isolate genome were calculated from tsv files. Nucleotide diversity at genomic positions was calculated using the Microsoft Excel software (https://www.microsoft.com/en-us/microsoft-365/excel; accessed on 18 May 2022, Microsoft Corporation, Redmond, WA, USA) with an in-house built file. It corresponded to the proportion of sequence reads that do not harbor the consensus (majority) nucleotide. Genome sequences obtained in the present study were submitted to the GISAID sequence database (https://www.gisaid.org/; accessed on 18 May 2022, 2008–2022, Freunde von GISAID e.V. Munich, Germany) [34] (see Supplementary Material: Supplementary Table S1).

### 4.3. Generation of Additional Sequence Reads

Sequencing of reverse-transcription-PCR-targeted regions: extraction of the RNA samples was carried out using the EZ1 Virus kit with the EZ1 Advanced XL instrument (Qiagen) following the manufacturer’s recommendations. PCR amplification of the 3 regions was amplified in a 25 μL total volume using the SuperScript III One-Step RT-PCR Kit (Invitrogen, Carlsbad, CA, USA), using primer concentrations of 200 nM per reaction. PCR were performed with following conditions: 50 °C for 25 min, 95 °C for 2 min, then 40 cycles including 15 s at 95 °C, 45 s at 60 °C, and 2 min at 68 °C. Sequences of PCR primers are provided in the Supplementary Material. Amplicons were sequenced with Nanopore technology on a GridION instrument (Oxford Nanopore Technologies Ltd., Oxford, UK), following the manufacturer’s instructions. Fastq files were processed as described above. Continuous reads overlapping signature mutations of distinct variants were filtered using SAMtools (v1.13; https://github.com/samtools/2008-2022 Genome Research Ltd., accessed on 18 May 2022) [45] combined with an in-house awk script. Reads were then filtered according to variant-specific nucleotide patterns using SAMtools combined with an in-house awk script. Groups of reads with same patterns of mutations were then visualized using the IGV software (https://software.broadinstitute.org/software/igv/; accessed on 18 May 2022, Broad Institute and the Regents of the University of California, USA) [48].

Metagenomic sequencing: nucleic acid extraction was performed with the EZ1 Virus kit with the EZ1 Advanced XL instrument (Qiagen) following the manufacturer’s recommendations, using 200 µL of sample and eluting in 60 µL of elution buffer. Reverse transcription was performed with all 60 µL of this solution using the TaqMan Reverse Transcription Reagent kit (Applied-Biosystems, Foster City, CA, USA), according to the manufacturer’s protocol under the following conditions: 10 min at 25 °C, 30 min at 48 °C, and 5 min at 95 °C. Then, 300 µL of obtained cDNA was transferred to a 1.5 mL Eppendorf LoBind tube (Eppendorf, Le Pecq, France). Second DNA strand was synthesized by adding a mix of 24 µL of Klenow Fragment DNA polymerase (New England Biolabs, Beverly, MA, USA), 66 µL of nuclease-free water, 45 µL of NEB Buffer 2 (New England Biolabs, Ipswich, MA, USA), and 15 µL of dNTPs working solution produced with 10 µL of each dNTP at a 100 mM concentration, and 60 µL of nuclease-free water (New England Biolabs, Ipswich, MA, USA). This mix was kept at 37 °C for one hour. A purification step consisted of adding 450 µL of magnetic CleanNGS beads for a 1:1 volume ratio (CleanNA, Waddinxveen, the Netherlands) then incubating them for 5 min in a magnetic support, and washing with 1000 µL of ethanol at 70%, before elution of the beads was performed in 50 µL of Tris EDTA 1X with centrifugation for 10 min at 300 rpm at room temperature. Subsequently, a DNA library was prepared with the DNA ligation sequencing kit SQK-LSK109 (Oxford Nanopore Technologies Ltd., Oxford, UK), and next-generation sequencing was performed with the Nanopore technology on a PromethION instrument (Oxford Nanopore Technologies Ltd., Oxford, UK). Each sample was sequenced on a different PromethION Flow cell R10.4 (Oxford Nanopore Technologies Ltd., Oxford, UK).

### 4.4. Phylogenetic Analysis Based on Whole and Partial Genome Sequences

Phylogenetic analyses were performed separately for the twelve genome sequences and the twelve spike gene sequences obtained from the nasopharyngeal samples or the culture supernatants. Sequences were aligned using MAFFT v.7 (Kazutaka Katoh and Daron M Standley, Osaka University, Japan) [49] with their 20 most similar hits identified with the BLAST tool (National Library of Medicine, Rockville Pike, MD, USA) [50] among SARS-CoV-2 genomes from our database, which contains sequences obtained from clinical samples collected between February 2020 and February 2022 [14,27]. Phylogeny reconstruction was performed using the IQ-TREE software with the GTR Model and 1000 ultrafast bootstrap repetitions (http://www.iqtree.org, Free Software Foundation, Boston, MA, USA) [51], and trees were visualized with iTOL (Interactive Tree of Life) (https://itol.embl.de/; accessed on 18 May 2022, EMBL 2022) [52] and MEGA X (v10.2.6; https://www.megasoftware.net/; accessed on 18 May 2022, 2007 Free Software Foundation) [53] softwares.

### 4.5. Virus Culture Isolation

Culture isolation was performed on Vero E6 cells, as previously described [33]. Briefly, 500 μL of nasopharyngeal swab fluid was passed through a centrifugal filter with a pore size of 0.22-μm (Merck Millipore, Darmstadt, Germany) before the inoculation of 100 µL of filtrate was performed in 4 wells of culture microplates with 96 wells that contained Vero E6 cells (ATCC CRL-1586) in Minimum Essential Medium culture medium, comprising 4% fetal calf serum and 1% glutamine. After a centrifugation step at 4000× *g*, culture microplates were incubated at 37 °C and were observed daily with an inverted microscope for evidence of cytopathogenic effect. We attempted, without success, to isolate the parental Alpha variant from respiratory samples collected in 2021 from the case-patient by selecting this virus through inoculating the respiratory samples with neutralizing serum from a convalescent individual previously infected by the B.1.160 variant, as previously reported [54], at equal volumes (50 µL of filtered respiratory sample and 50 µL of serum).

## Figures and Tables

**Table 1:** Genome sequences obtained from the sequential nasopharyngeal samples of the case-patient.

GISAID Identifier	Sampling Date	Time from Diagnosis (Days)	Next-Generation Sequencing Technology, Instrument
EPI_ISL_6332079	18 September 2020	0	Illumina, NovaSeq
EPI_ISL_10816743	5 May 2021	229	Illumina, NovaSeq
EPI_ISL_11030507	21 June 2021	276	Illumina, NovaSeq
EPI_ISL_10816731	5 July 2021	290	Illumina, NovaSeq
EPI_ISL_10816742	12 August 2021	328	Nanopore, GridION
EPI_ISL_10816744	1 October 2021	378	Illumina, NovaSeq
EPI_ISL_10816733	24 November 2021	432	Illumina, NovaSeq

See also Supplementary Table S3a.

**Table 2:** Genome sequences obtained from the culture supernatants.

GISAID Identifier	Sampling Date of the Nasopharyngeal Sample	Time to Cytopathic Effect (Days)	Next-Generation Sequencing Technology, Instrument
EPI_ISL_10816730	5 May 2021	8	Illumina, NovaSeq
EPI_ISL_10816732	14 June 2021	4	Illumina, NovaSeq
EPI_ISL_10816734	12 August 2021	4	Illumina, NovaSeq
EPI_ISL_10816735	24 November 2021	5	Illumina, NovaSeq
EPI_ISL_10816738	2 December 2021	7	Illumina, NovaSeq

See also Supplementary Table S3b.

## Data Availability

The dataset generated and analyzed during the current study is available in the GISAID sequence database (https://www.gisaid.org/; accessed on 18 May 2022, 2008–2022, Freunde von GISAID e.V. Munich, Germany) [34].

## Supplementary Materials

The following supporting information can be downloaded at: https://www.mdpi.com/article/10.3390/v14061266/s1. Additional Methods, Results, Figure S1: Virological follow-up by real-time reverse transcription PCR (qPCR) targeting SARS-CoV-2 RNA for the patient, Figure S2: Majority nucleotides and nucleotide diversity for sequences obtained from the respiratory samples and the culture supernatant at nucleotide positions of the SARS-CoV-2 genome that harbor signature mutations of the B.1.160 or Alpha variants and at any other positions that harbor mutations (a), and sequencing depth at nucleotide positions (b), Figure S3: Detailed content of amplicons corresponding to positions 3100–4570 of the SARS-CoV-2 genome and retrieved from respiratory samples collected on 5 May 2021, Figure S4: Detailed content of amplicons corresponding to positions 3100–4570 of the SARS-CoV-2 genome and retrieved from respiratory samples collected on 12 August 2021, Figure S5: Detailed content of amplicons corresponding to positions 24,880–29,010 of the SARS-CoV-2 genome and retrieved from respiratory samples collected on 12 August 2021, Table S1: List of GISAID identifiers for sequences used in the present study, and Table S2: Sampling time and location for sequences used in the present study, Table S3: Identifiers of raw data deposited in the European Bioinformatics Institute (EMBL-EBI) sequence database (https://www.ebi.ac.uk/; accessed on 18 May 2022), and References, are provided in the Supplementary Material.
