# Supplementary material for: Sequential Appearance and Isolation of a SARS-CoV-2 Recombinant between Two Major SARS-CoV-2 Variants in a Chronically Infected Immunocompromised Patient
Source: Viruses. 2022 Jun 10;14(6):1266. doi: 10.3390/v14061266 (PMC9227898; doi:10.3390/v14061266)
Supplement: Supplementary file 1 [file viruses-14-01266-s001.zip › viruses-1667331-supplementary.pdf]

Article

Sequential Appearance and Isolation of a SARS-CoV-2 Recombinant between Two Major SARS-CoV-2 Variants in a Chronically Infected Immunocompromised Patient

Emilie Burel [1,2,†], Philippe Colson [1,2,3,†], Jean-Christophe Lagier [1,2,3], Anthony Levasseur [1,2], Marielle Bedotto [1], Philippe Lavrard-Meyer [1,2,3], Pierre-Edouard Fournier [1,2,4], Bernard La Scola [1,2,3,*] and Didier Raoult [1,2,*]

[1] IHU Méditerranée Infection, 19-21 Boulevard Jean Moulin, 13005 Marseille, France; burel.emilie@hotmail.fr (E.B.); philippe.colson@univ-amu.fr (P.C.); jean-christophe.lagier@univ-amu.fr (J.-C.L.); anthony.levasseur@univ-amu.fr (A.L.); marielle.bedotto@gmail.com (M.B.); philippe.lavrard-meyer@ap-hm.fr (P.L.-M.); pierre-edouard.fournier@univ-amu.fr (P.-E.F.)

[2] Microbes Evolution Phylogeny and Infections (MEPHI), Institut de Recherche Pour le Développement (IRD), Aix-Marseille University, 27 Boulevard Jean Moulin, 13005 Marseille, France

[3] Assistance Publique-Hôpitaux de Marseille (AP-HM), 264 rue Saint-Pierre, 13005 Marseille, France

[4] Vecteurs-Infections Tropicales et Méditerranéennes (VITROME), Institut de Recherche Pour le Développement (IRD), Aix-Marseille University, 27 Boulevard Jean Moulin, 13005 Marseille, France

* Correspondence: bernard.la-scola@univ-amu.fr (B.L.S.); didier.raoult@univ-amu.fr (D.R.); Tel.: +33-413-732-401 (B.L.S. & D.R.)

† These authors contributed equally to this work.

1. SUPPLEMENTARY METHODS

Nasopharyngeal samples from the immunocompromised patient were tested in our university hospital institute (Méditerranée Infection; https://www.mediterranee-infection.com/) from September 2020 to December 2021 for SARS-CoV-2 infection by real-time reverse-transcription-PCR (qPCR) using the BGI real-time fluorescent RT-PCR assay (BGI Genomics, Shanghai Fosun Long March Medical Science Co., Ltd., Shenzhen, China) or the NeuMoDx SARS-CoV-2 assay (NeuMoDx, Ann Arbor, Michigan). Cycle threshold values for qPCR positivity was 35. Outside our institute (medical biology laboratory Alphabio, hôpital Européen, France), SARS-CoV-2 qPCR was performed using the BD MAX System (Becton Dickinson, Sparks, MD, USA).

2. SUPPLEMENTARY RESULTS

2.1. Virological follow-up by SARS-CoV-2 real-time reverse-transcription-PCR (qPCR)

SARS-CoV-2 qPCR performed on nasopharyngeal samples was positive between September 2020 and January 2021, then negative once in February 2021 but positive again on the next sample tested in April 2021. Between May 2021 and August 2021, qPCR remained almost always positive being only transiently negative for ≤3 days. Then qPCR was consistently positive when performed between October and December 2021.

2.2. Nucleotide diversity at positions harboring signature mutations of the B.1.160 or Alpha variants

Regarding the SARS-CoV-2 genomes obtained from the respiratory samples, which were obtained with the Illumina technology except for the sample collected in August 2021 for which the genome was obtained with the Nanopore technology, nucleotide diversity at the 35 positions harboring signature mutations of the B.1.160 or Alpha variants differed according to the time of sample collection. It was low in September 2020, with a mean (±standard deviation) value of 0.2±0.5%, increased to reach high values in May 2021 (20.1±16.4%), June 2021 (14.7±14.3%), July 2021 (19.8±18.2%), and August 2021

(13.2±8.9%), then decreased to low values again in October 2021 (1.1±3.9%) and November 2021 (3.1±6.8%) (Figure 2 of the main text). The low nucleotide diversity observed for genomes obtained in October and November 2021 indicated that the mosaicism consisting in the presence of signature mutations of the two variants was not explained by a co-infection by these two variants or by a contamination of the samples prior or during the next-generation sequencing procedure.

Regarding the genomes obtained from the culture supernatants, which were all obtained with the Illumina technology, nucleotide diversity at positions harboring signature mutations of the B.1.160 or Alpha variants was low, indicating that these genomes, including those recombinants, were generated from a single viral isolate. Indeed, mean nucleotide diversity ranged between 0.6±2.0% and 4.3±6.4% (Figure 2 of the main text). The viral genomes obtained from the culture of the respiratory samples collected in November and December 2021 were highly similar to the viral genome obtained directly from the respiratory sample collected in November 2021.

2.3. Generation of additional sequence reads

Sequencing of reverse-transcription-PCR-targeted regions: We attempted to generate sequence reads that are hybrids of the B.1.160 and Alpha variants from the respiratory sample collected in August 2021 by PCR amplification of the regions overlapping recombination sites then next-generation sequencing with the Oxford Nanopore Technology (ONT) on a GridION instrument (Oxford Nanopore Technologies Ltd., Oxford, United Kingdom). Amplicons corresponding to positions 3,100-4,570 (in reference to the genome of the Wuhan-Hu-1 isolate GenBank accession no. NC_045512.2) were amplified using the following primers (in 5′-3′ orientation): Forward: TTCAACCTGAAGAAGAGCAA; reverse: TGGCATTGTAACAAGAGTTT. Amplicons corresponding to positions 24,813-29,074 were amplified using the following primers: Forward: ATGGAAAAGCACACTTTCCT; reverse: GCTTTAGTGGCAGTACGTTT. For the amplicons corresponding to positions 3,100-4,570, 56% and 87% of the reads were chimeras harboring both Alpha (C3267U) and Marseille-4 (C4543U) signature mutations from the respiratory samples collected in May 5th and in August 12th, 2021, respectively (Supplementary Material: Figures S3, S4). For the amplicon corresponding to positions 24,813-29,074, 70% of the reads were hybrids harboring Alpha (G24914C) and B.1.160 (G25563U, C25710U, C26735U, U26876C and G28975C) mutations, and 24% were hybrids harboring Alpha mutation G24914C and B.1.160 mutation G28975C (Supplementary Material: Figure S5).

Metagenomic sequencing: Next-generation sequencing was also carried out using a metagenomic approach with the Nanopore technology on a GridION instrument (Oxford Nanopore Technologies Ltd.) to attempt obtaining long sequence reads and detect additional reads that are hybrids of the B.1.160 and Alpha variants. For the first recombination site, four reads with a length ranging between 1,855-11,979 nucleotides were obtained, which harbored signature mutations of the Alpha (C3267U) and B.1.160 (C4543U) variants. For the third recombination site, four reads with a length comprised between 1,486-8,143 nucleotides were obtained that harbored signature mutations of the Alpha (G24914C) and B.1.160 (C25563U) variants (reads are available from URL: https://www.mediterranee-infection.com/sars-cov-2-recombinant/).

2.4. Viral culture

Cytopathic effects were observed between 4 and 8 days after the inoculation of the nasopharyngeal samples on Vero E6 cells as previously described [1] (Table 2 of the main text).

2.5. Phylogenetic analyses based on SARS-CoV-2 genomes and spike gene sequences

Phylogeny reconstructions were performed based on viral genomes or spike genes, which included sequences obtained directly from the nasopharyngeal samples and from the cultures, and their best BLAST hits from the IHU Méditerranée Infection sequence database that were classified as B.1.160 or Alpha variants [2]. The genome obtained from

the nasopharyngeal sample collected in September 2020 was clustered with genomes of the B.1.160 variant, apart from the other genomes obtained from the case-patient (Figure 3a of the main text). Other genomes obtained from the nasopharyngeal samples collected since May 2021 and those obtained from cultures of the nasopharyngeal samples collected since May 2021 were also clustered with genomes of the B.1.160 variant, but they were clustered together, apart from the other genomes. The Alpha variant genomes were clustered apart from all other genomes. Spike gene phylogeny showed the clusterisation with sequences of the B.1.160 variant of the sequences obtained from the case-patient either directly or post-culture from samples collected between September 2020 and June 2021 (Figure 3b of the main text). In contrast, sequences obtained from the case-patient either directly or post-culture from samples collected since August 2021 were clustered with Alpha variant sequences.

3. SUPPLEMENTARY FIGURES

Supplementary Figure S1. Virological follow-up by real-time reverse transcription PCR (qPCR) targeting SARS-CoV-2 RNA for the patient.

Time points of collection of nasopharyngeal samples used for direct next-generation sequencing are indicated by *; time points of collection of samples from which positive culture was obtained leading to next-generation sequencing from the culture supernatant are indicated by #.

Nasopharyngeal samples from the immunocompromised patient were tested in our university hospital institute (Méditerranée Infection; https://www.mediterranee-infection.com/) from September 2020 to December 2021 for SARS-CoV-2 infection by real-time reverse-transcription-PCR (qPCR) using the BGI real-time fluorescent RT-PCR assay (BGI Genomics, Shanghai Fosun Long March Medical Science Co., Ltd., Shenzhen, China) or the NeuMoDx SARS-CoV-2 assay (NeuMoDx, Ann Arbor, Michigan). Cycle threshold values for qPCR positivity was 35. Outside our institute (medical biology laboratory Alphabio, hôpital Européen, France), SARS-CoV-2 qPCR was performed using the BD MAX System (Becton Dickinson, Sparks, MD, USA).

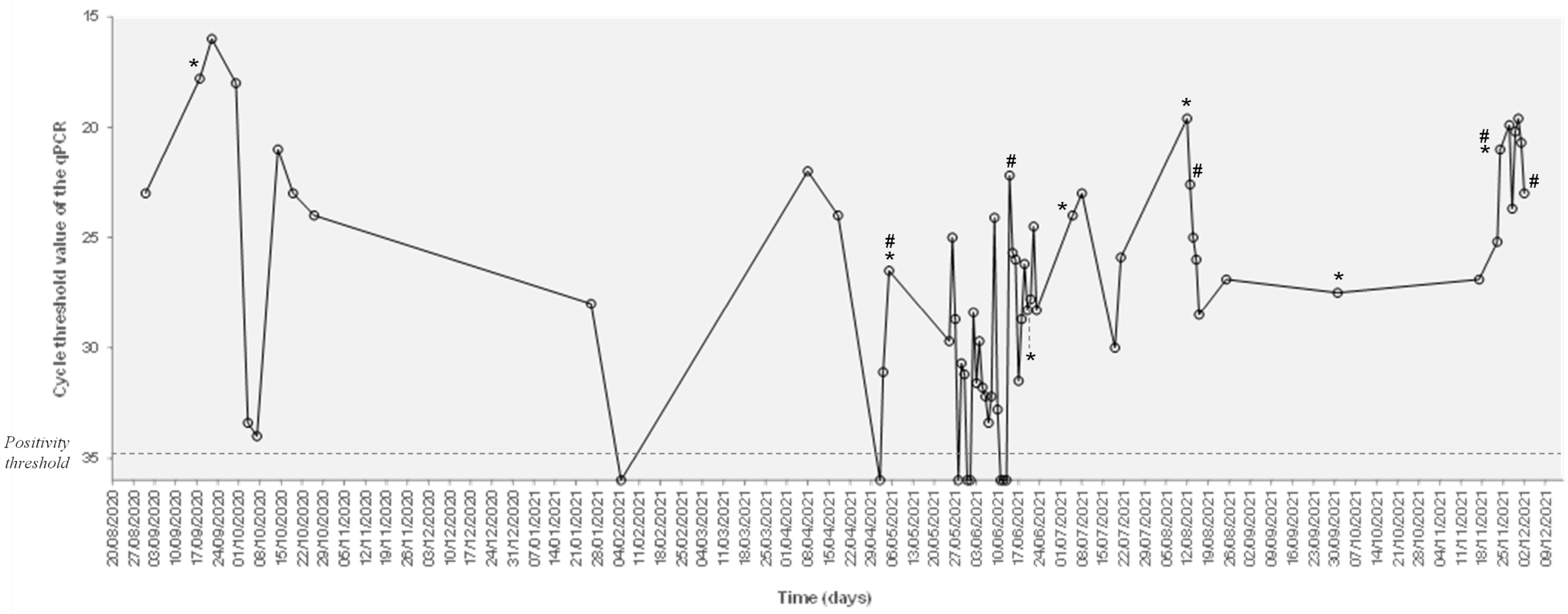
Cycle threshold value of the qPCR
15
20
25
30
35
Positivity threshold
Time (days)
20/08/2020
27/08/2020
03/09/2020
10/09/2020
17/09/2020
24/09/2020
01/10/2020
08/10/2020
15/10/2020
22/10/2020
29/10/2020
05/11/2020
12/11/2020
19/11/2020
26/11/2020
03/12/2020
10/12/2020
17/12/2020
24/12/2020
31/12/2020
07/01/2021
14/01/2021
21/01/2021
28/01/2021
04/02/2021
11/02/2021
18/02/2021
25/02/2021
04/03/2021
11/03/2021
18/03/2021
25/03/2021
01/04/2021
08/04/2021
15/04/2021
22/04/2021
29/04/2021
06/05/2021
13/05/2021
20/05/2021
27/05/2021
03/06/2021
10/06/2021
17/06/2021
24/06/2021
01/07/2021
08/07/2021
15/07/2021
22/07/2021
29/07/2021
05/08/2021
12/08/2021
19/08/2021
26/08/2021
02/09/2021
09/09/2021
16/09/2021
23/09/2021
30/09/2021
07/10/2021
14/10/2021
21/10/2021
28/10/2021
04/11/2021
11/11/2021
18/11/2021
25/11/2021
02/12/2021
09/12/2021

Supplementary Figure S2. Majority nucleotides and nucleotide diversity for sequences obtained from the respiratory samples and the culture supernatant at nucleotide positions of the SARS-CoV-2 genome that harbor signature mutations of the B.1.160 or Alpha variants and at any other positions that harbor mutations (a), and sequencing depth at nucleotide positions (b).

Del, nucleotide deletion.

Nucleotide positions are in reference to the genome of the Wuhan-Hu-1 isolate GenBank accession no. NC_045512.2. Nucleotide diversity is the proportion of sequence reads that do not harbor the consensus (majority) nucleotide.

Supplementary Figure S2a.

Nucleotide positions	Genes	Wuhan-Hu-1 isolate genome	Signature mutations in B.1.160/Marseille-4 genomes	Signature mutations in Alpha genomes	Majority nucleotides											
					Nasopharyngeal samples							Culture supernatants				
					September 18th, 2020	May 5th, 2021	June 21st, 2021	July 5th, 2021	August 12th, 2021	October 1st, 2021	November 24th, 2021	May 5th, 2021	June 14th, 2021	August 13th, 2021	November 24th, 2021	December 2nd, 2021
241	ORF 1a	C	U	U	U	U	U	U	U	U	U	U	U	U	U	U
629		C	-	-	-	U	U	U	U	U	U	-	-	U	U	U
696		A	-	-	-	-	-	U	U	U	U	-	-	U	U	U
913		C	-	U	-	-	-	U	U	U	U	-	-	U	U	U
981		C	-	-	-	-	-	-	-	-	U	-	-	-	-	-
1192		A	-	-	-	-	-	-	-	G	-	-	-	-	-	-
1677		A	-	-	-	-	-	-	-	-	-	-	-	-	C	-
1683		U	-	-	-	-	-	-	-	-	C	-	-	-	-	C
1895		G	-	-	-	-	-	-	-	A	-	-	-	-	-	-
1978		U	-	-	-	-	-	-	-	C	-	-	-	-	-	-
2509		C	-	-	-	-	-	-	-	-	-	-	-	-	U	-
2594		C	-	-	-	-	-	-	-	-	U	-	-	-	U	U
2706		C	-	-	-	U	U	-	-	-	U	U	U	-	U	U
3037		C	U	U	U	U	U	U	U	U	U	U	U	U	U	U
3193		A	-	-	-	-	-	G	G	G	-	-	-	G	-	-
3224		A	-	-	-	-	-	-	-	G	-	-	-	-	-	-
3267		C	-	U	-	-	-	U	U	U	-	-	-	U	-	-
3340		G	-	-	-	-	-	-	-	-	-	-	-	-	U	-
3393		C	-	-	-	-	U	-	-	-	-	U	U	-	-	-
4543		C	U	-	U	U	U	U	U	U	U	U	U	U	U	U
4872		A	-	-	-	G	G	-	-	-	-	G	G	-	-	-
4878		C	-	-	-	U	U	U	U	U	U	U	U	U	U	U
4893		C	-	-	-	-	-	U	U	U	U	-	-	U	U	U
4904		G	-	-	-	-	A	-	-	-	-	A	A	-	-	-
5278		U	-	-	-	-	-	-	-	-	-	-	-	-	C	-
5629		G	U	-	U	U	U	U	U	U	U	U	U	U	U	U
5648		A	-	-	-	-	-	-	-	-	C	-	-	-	C	C
5986		C	-	U	-	-	-	-	-	-	-	-	-	U	-	-
6629		C	-	-	-	-	U	-	-	-	-	-	U	-	-	-
6633		C	-	-	-	U	U	U	U	U	U	U	U	U	U	U
7113		C	-	-	-	-	-	-	-	-	U	-	-	-	-	U
7185		U	-	-	-	C	C	C	C	C	C	C	C	C	C	C
7279		C	-	-	-	-	-	-	-	-	U	-	-	-	-	-
8191		U	-	-	-	-	-	-	-	-	-	-	-	A	-	-
8770		C	-	-	-	U	-	-	-	-	-	-	-	-	-	-
8950		C	-	-	-	-	-	-	-	-	-	-	U	-	-	-
9042		C	-	-	-	U	U	U	U	U	U	U	U	U	U	U
9120		C	-	-	-	U	U	U	U	U	U	U	U	U	U	U
9501		C	-	-	-	U	-	U	U	U	U	U	U	U	U	U
9526		G	U	-	U	U	U	U	U	U	U	U	U	U	U	U
9626		U	-	-	-	C	-	-	-	-	-	-	-	-	-	-
9783		G	-	-	-	-	-	-	C	-	-	-	-	-	-	-
9792		A	-	-	-	-	C	-	-	-	-	-	-	-	-	-
9811		C	-	-	-	-	-	-	-	-	U	-	-	-	U	U
9972		A	-	-	-	-	-	-	-	-	G	-	-	-	-	-
11083		G	-	-	-	-	-	-	-	-	-	U	-	-	-	-
11208		C	-	-	-	U	U	U	U	U	U	U	U	U	U	U
11365		G	-	-	-	-	-	-	-	-	U	-	-	-	-	U
11497		C	U	-	U	U	U	U	U	U	U	U	U	U	U	U
12459		C	-	-	-	-	U	-	-	-	-	-	U	-	-	-
12592		U	-	-	-	-	C	-	-	-	-	-	C	-	-	-

Nucleotide positions	Nucleotide diversity (%)											
	Nasopharyngeal samples							Culture supernatants				
	September 18th, 2020	May 5th, 2021	June 21th, 2021	July 5th, 2021	August 12th, 2021	October 1st, 2021	November 24th, 2021	May 5th, 2021	June 14th, 2021	August 13th, 2021	November 24th, 2021	December 2nd, 2021
241	0,0	0,0	0,0	0,1	10,6	0,0	0,1	0,0	0,1	0,0	0,0	0,4
629	0,2	45,5	13,2	25,1	28,4	0,1	1,0	6,4	0,1	0,2	0,1	0,3
696	0,0	37,5	29,6	32,0	4,5	0,2	0,1	4,9	0,0	0,1	0,5	0,2
913	0,0	33,9	26,8	35,9	10,0	0,1	0,1	9,0	0,1	0,1	0,1	0,3
981	0,0	0,0	0,1	0,0	4,0	0,0	14,0	0,0	0,2	0,1	0,4	0,4
1192	0,2	0,0	0,1	0,2	2,6	33,3	0,0	0,0	0,0	0,1	0,1	0,1
1677	0,0	0,0	0,0	0,0	2,3	0,0	15,0	0,1	0,1	0,1	0,3	0,0
1683	0,0	0,0	0,0	0,0	1,4	0,0	18,5	0,1	0,1	0,0	0,1	0,1
1895	0,5	0,0	0,0	0,0	6,4	0,0	0,0	0,0	0,0	0,5	0,0	0,3
1978	0,1	0,0	0,0	0,0	1,0	12,7	0,2	0,1	0,2	0,0	0,2	0,3
2509	0,5	0,0	0,0	0,0	3,4	0,0	7,4	0,0	0,0	0,0	0,8	0,0
2594	0,0	0,0	0,0	0,0	2,2	0,0	0,0	0,1	0,0	0,0	0,1	0,2
2706	0,1	43,6	25,0	28,1	5,4	0,0	0,1	4,3	0,1	0,0	0,2	0,0
3037	0,3	0,3	1,3	0,0	20,8	0,0	0,1	0,0	0,1	0,0	0,4	0,1
3193	0,3	0,0	25,5	33,4	21,2	0,9	0,0	0,0	0,0	2,6	0,0	0,6
3224	0,0	0,0	0,0	0,0	6,3	0,1	0,1	0,0	0,0	0,0	0,0	0,0
3267	0,0	28,8	23,7	39,3	6,8	0,0	0,0	2,4	0,1	0,2	0,4	0,1
3340	0,0	16,8	0,0	0,0	2,3	0,1	12,7	0,1	0,1	0,0	0,0	0,3
3393	0,3	28,7	43,4	20,8	4,6	0,0	0,2	2,4	0,0	0,0	0,0	0,1
4543	0,1	0,0	0,0	0,0	7,8	0,0	0,1	0,3	0,2	0,2	0,8	0,1
4872	0,1	0,0	12,5	37,1	2,9	0,0	0,0	0,0	0,1	0,0	0,1	0,1
4878	0,0	0,0	0,0	0,0	6,6	0,0	0,2	0,1	0,1	0,1	0,2	0,2
4893	0,1	0,0	27,3	43,3	5,2	0,6	0,0	0,1	0,0	0,2	0,1	0,2
4904	0,0	10,5	30,0	47,9	1,7	0,0	0,0	4,8	0,0	0,0	0,2	0,1
5278	0,9	0,0	0,0	0,0	2,1	0,0	14,6	0,0	0,0	0,0	8,0	0,0
5629	2,8	0,5	1,6	0,2	11,4	0,6	0,0	0,8	1,9	0,9	5,3	1,5
5648	0,1	0,1	0,0	0,0	1,6	0,0	0,1	0,1	0,0	0,0	0,2	0,1
5986	0,0	0,0	0,0	0,0	10,3	0,0	0,0	0,0	0,0	18,4	0,0	0,1
6629	0,0	0,0	37,7	15,6	5,6	0,0	0,0	0,0	0,3	0,3	0,1	0,1
6633	0,0	0,2	0,0	0,0	11,0	0,1	0,0	0,0	0,1	0,3	0,1	0,2
7113	0,0	0,0	/	0,0	3,4	0,0	15,5	0,0	0,0	0,0	0,3	1,5
7185	0,1	0,0	0,0	0,0	10,9	0,0	0,0	0,1	0,0	0,0	0,4	0,1
7279	0,1	0,0	0,0	2,7	2,5	0,0	31,3	0,2	0,3	0,5	0,3	0,5
8191	0,1	0,0	0,0	0,0	12,0	0,2	0,2	0,1	0,0	16,0	0,2	0,0
8770	0,0	47,2	0,0	0,0	10,6	0,0	0,1	3,1	0,1	0,0	0,7	0,1
8950	0,1	0,0	48,1	14,9	1,6	0,1	0,1	0,2	0,1	0,1	0,2	0,1
9042	0,0	0,1	0,1	0,2	17,7	0,0	0,1	0,1	0,5	0,1	0,1	0,3
9120	0,0	0,0	0,0	0,0	8,6	0,0	0,3	0,0	0,0	0,1	0,2	0,3
9501	0,0	0,0	/	0,0	9,1	0,0	0,0	0,6	2,6	0,4	0,5	2,2
9526	0,1	0,0	0,0	0,0	4,6	0,0	0,8	1,2	0,2	0,2	0,0	0,3
9626	0,0	50,0	/	0,0	15,5	0,0	0,0	0,3	0,1	0,0	0,0	0,5
9783	0,0	0,0	/	0,0	35,0	0,0	0,0	0,0	0,0	0,0	0,0	0,2
9792	0,0	16,7	0,0	0,0	2,2	0,0	0,0	1,2	0,0	0,0	0,0	0,0
9811	0,1	0,0	0,0	0,0	6,1	0,0	2,9	0,0	0,1	0,2	0,7	0,6
9972	0,0	0,0	0,0	0,0	5,7	0,0	21,6	0,0	0,0	0,2	0,0	0,1
11083	1,0	1,1	0,0	0,0	2,2	0,0	0,7	27,1	0,1	0,1	0,7	0,5
11208	0,0	0,0	0,2	0,1	4,6	0,2	0,0	0,2	0,1	0,0	0,2	0,1
11365	0,0	0,0	0,0	0,0	2,3	0,0	19,2	0,1	0,0	0,0	0,1	0,1
11497	0,4	0,0	0,0	0,3	2,5	0,4	0,0	0,1	0,3	0,2	0,1	0,2
12459	0,4	0,0	45,8	20,6	3,1	0,0	0,1	0,3	0,1	0,4	0,2	0,3
12592	0,0	0,2	39,0	23,5	2,1	0,0	0,0	0,1	0,1	0,0	0,1	0,1

Supplementary Figure S2a (continued)

Nucleotide positions	Genes	Wuhan-Hu-1 isolate genome	Signature mutations in B.1.160/Marseille-4 genomes	Signature mutations in Alpha genomes	Majority nucleotides												Nucleotide diversity (%)											
					Nasopharyngeal samples							Culture supernatants					Nasopharyngeal samples							Culture supernatants				
					September 18th, 2020	May 5th, 2021	June 21st, 2021	July 5th, 2021	August 12th, 2021	October 1st, 2021	November 24th, 2021	May 5th, 2021	June 14th, 2021	August 13th, 2021	November 24th, 2021	December 2nd, 2021	September 18th, 2020	May 5th, 2021	June 21th, 2021	July 5th, 2021	August 12th, 2021	October 1st, 2021	November 24th, 2021	May 5th, 2021	June 14th, 2021	August 13th, 2021	November 24th, 2021	December 2nd, 2021
13506	ORF 1b	C	-	-	-	U	U	U	U	U	U	U	U	U	U	U	0,0	0,0	0,0	0,0	13,9	0,0	0,0	0,0	0,1	0,0	0,1	0,1
13517		C	-	-	-	-	-	-	-	U	-	-	-	-	-	-	0,3	0,0	0,0	0,0	3,6	16,7	0,1	0,4	0,1	0,6	0,1	0,2
13860		C	-	-	-	-	-	U	U	U	-	-	-	U	-	-	0,2	0,1	24,2	35,4	3,9	0,1	0,1	0,1	0,2	0,0	0,1	0,2
13936		G	-	-	-	-	-	-	-	-	-	-	-	-	-	U	0,0	0,0	0,1	0,0	4,3	0,3	0,0	0,1	0,0	0,0	0,1	3,0
13993		G	U	-	U	U	U	U	U	U	U	U	U	U	U	U	0,1	0,1	0,0	0,1	5,4	0,1	0,0	0,0	0,1	0,0	0,3	0,1
14408		C	U	U	U	U	U	U	U	U	U	U	U	U	U	U	0,1	0,1	0,0	0,1	11,0	0,1	0,4	0,1	0,3	0,0	0,3	0,2
15766		G	U	-	U	U	U	U	U	U	U	U	U	U	U	U	0,1	0,3	0,0	0,1	9,2	0,0	0,0	0,0	0,1	0,0	0,1	0,1
16128		A	-	-	-	-	-	-	-	-	-	-	-	-	G	-	0,2	0,0	0,1	0,1	2,3	0,0	13,9	0,0	0,0	0,0	12,1	0,0
16675		C	-	-	-	U	U	U	U	U	U	U	U	U	U	U	0,1	0,2	0,0	0,2	13,2	0,1	0,0	0,1	0,7	0,5	0,2	0,1
16751		C	-	-	-	U	U	U	U	U	U	U	U	U	U	U	7,0	0,0	0,0	0,2	11,2	0,4	0,6	0,3	0,9	0,0	0,6	0,0
16889		A	G	-	G	G	G	G	G	G	G	G	G	G	G	G	0,1	0,0	0,0	0,0	7,7	0,0	0,2	0,0	0,1	0,0	0,3	0,1
17019		G	U	-	U	U	U	U	U	U	U	U	U	U	U	U	0,0	0,0	0,0	0,0	15,6	0,0	0,0	0,3	0,2	0,4	0,1	0,1
17122		G	-	-	-	-	-	-	-	-	U	-	-	-	-	U	0,1	0,0	0,0	0,0	11,9	0,1	16,0	0,0	0,1	0,1	0,1	0,1
17125		C	-	-	-	-	-	U	U	U	-	-	-	U	-	-	0,1	0,0	18,9	43,4	18,4	0,1	0,0	0,1	0,0	0,1	0,2	0,2
17251		C	-	-	-	U	U	U	U	U	U	U	U	U	U	U	0,0	0,3	0,1	0,1	11,1	0,2	0,1	0,2	0,1	0,1	0,5	0,5
17721		G	-	-	-	-	-	-	-	-	-	-	-	-	U	-	0,1	0,0	0,0	0,0	5,0	0,0	11,7	0,1	0,1	0,1	0,2	0,3
17738		C	-	-	-	-	-	-	-	U	-	-	-	-	-	-	0,0	0,0	0,0	0,1	9,3	1,0	0,0	0,1	0,1	0,1	0,2	0,1
18023		A	-	-	-	-	-	-	-	-	G	-	-	-	-	-	0,0	0,2	0,1	0,1	0,9	0,2	22,3	0,2	0,0	0,1	0,5	0,4
18877		C	U	-	U	-	U	U	U	U	-	U	U	U	-	-	0,0	47,0	0,0	0,0	6,8	0,0	0,1	2,7	0,2	0,0	0,2	0,1
19186		C	-	-	-	-	-	-	-	-	-	-	-	-	-	-	0,1	0,1	0,1	0,0	4,5	0,0	0,5	0,3	0,0	0,2	44,5	0,1
19547		C	-	-	-	-	-	-	-	-	-	-	-	U	-	-	0,0	0,0	0,0	0,0	4,9	0,0	0,0	0,0	0,0	15,2	0,1	0,3
19947		G	-	-	-	-	-	-	U	-	-	-	-	-	-	-	0,0	0,0	0,0	0,0	59,4	0,0	0,2	0,0	0,2	0,0	0,4	0,1
20930		C	-	-	-	-	-	-	-	-	-	U	-	-	-	-	0,1	18,5	0,0	0,0	5,8	0,1	0,0	6,2	0,1	0,0	0,2	0,1
21219		C	-	-	-	-	-	-	-	-	U	-	-	-	-	-	0,0	0,0	0,0	0,0	0,0	0,0	30,1	0,1	0,0	0,0	0,2	0,1
21232		G	-	-	-	-	-	-	-	-	-	-	-	-	U	-	0,1	0,0	0,0	0,0	0,0	0,0	19,3	0,0	0,1	0,0	0,3	0,3
21575	SPIKE	C	-	-	-	-	U	-	-	-	-	U	U	-	-	-	1,2	44,4	42,4	14,4	8,2	0,0	0,5	4,4	1,2	5,4	0,1	0,5
21765		U	-	Del	-	-	-	-	Del	Del	Del	-	-	Del	Del	Del	0,1	14,7	11,0	38,3	39,2	22,6	22,9	2,1	0,0	21,8	23,7	21,2
21766		A	-	Del	-	-	-	-	Del	Del	Del	-	-	Del	Del	Del	0,0	16,2	11,8	44,0	10,9	1,6	1,1	2,2	0,0	1,2	1,6	1,0
21767		C	-	Del	-	-	-	-	Del	Del	Del	-	-	Del	Del	Del	0,1	15,4	11,1	42,8	19,6	0,0	0,0	2,1	0,0	0,0	0,1	0,0
21768		A	-	Del	-	-	-	-	Del	Del	Del	-	-	Del	Del	Del	0,0	15,4	11,2	43,1	10,4	0,0	0,0	2,1	0,0	0,0	0,1	0,1
21769		U	-	Del	-	-	-	-	Del	Del	Del	-	-	Del	Del	Del	0,0	15,6	11,5	43,7	13,7	0,2	0,2	2,1	0,1	0,2	0,5	0,5
21770		G	-	Del	-	-	-	-	Del	Del	Del	-	-	Del	Del	Del	0,1	18,2	15,2	47,6	9,6	5,6	5,6	3,6	0,0	7,2	6,4	6,5
21991		U	-	Del	-	-	Del	Del	Del	Del	Del	-	Del	Del	Del	Del	0,0	20,3	17,3	18,6	22,1	1,3	4,3	0,1	2,1	2,5	0,7	1,4
21992		U	-	Del	-	-	Del	Del	Del	Del	Del	-	Del	Del	Del	Del	0,1	20,4	16,9	18,1	20,3	1,3	5,6	0,1	2,1	2,5	2,0	2,1
21993		A	-	Del	-	-	Del	Del	Del	Del	Del	-	Del	Del	Del	Del	0,1	20,6	16,7	17,7	17,8	3,7	18,9	0,1	11,5	7,4	10,7	12,6
22992		G	A	-	A	A	/	-	-	-	-	A	A	-	-	-	0,3	33,3	/	20,6	4,8	0,0	0,0	5,5	0,1	0,0	0,2	0,3
23063		A	-	U	-	-	-	U	U	U	U	-	-	U	U	U	0,0	50,0	33,3	27,1	12,4	0,0	0,0	6,7	0,1	0,0	0,4	0,4
23188		C	-	-	-	-	-	-	-	-	-	U	-	-	-	-	0,0	0,0	0,0	0,0	2,3	0,0	0,1	7,3	0,0	0,1	0,2	0,1
23271		C	-	A	-	-	-	A	A	A	A	-	-	A	A	A	0,2	15,7	20,0	44,2	4,2	0,0	0,1	5,1	0,0	0,3	0,2	0,2
23277		C	-	-	-	-	-	-	-	-	-	-	-	-	-	U	0,0	0,0	0,0	0,0	7,9	0,0	0,2	0,2	0,0	0,2	0,1	0,3
23403		A	G	G	G	G	G	G	G	G	G	G	G	G	G	G	0,2	0,0	0,0	0,0	17,4	0,1	0,0	0,1	0,1	0,0	0,1	0,2
23415		C	-	-	-	-	-	-	-	-	A	-	-	-	-	A	0,1	0,0	0,0	0,0	3,6	1,3	14,0	0,2	0,0	0,1	0,1	0,2
23525		C	-	-	-	-	-	-	-	-	-	-	-	-	-	U	0,0	0,0	0,0	0,0	2,4	0,0	0,0	0,1	0,1	0,0	0,2	0,2
23598		A	-	-	-	-	-	-	-	-	-	G	-	-	-	-	0,1	0,0	0,1	0,0	1,6	0,0	0,1	49,0	0,1	0,8	0,4	0,0
23604		C	-	A	-	-	-	A	A	A	A	A	-	A	A	A	0,1	24,8	25,6	37,8	6,0	0,0	0,1	32,7	0,0	0,9	0,6	0,2
23709		C	-	U	-	-	-	U	U	U	U	-	-	U	U	U	0,1	27,5	28,1	37,8	7,8	0,0	0,1	3,5	0,1	0,1	0,1	0,1
23948		G	-	-	-	C	C	-	-	-	-	C	C	-	-	-	0,0	36,9	37,9	29,0	2,2	0,0	0,2	4,1	0,0	0,0	0,3	0,4
24023		C	-	-	-	U	U	-	-	-	-	U	U	-	-	-	0,0	43,5	43,5	27,4	2,0	0,0	0,4	5,7	0,3	0,2	0,2	0,7
24506		U	-	G	-	-	-	G	G	G	G	-	-	G	G	G	0,0	31,2	25,0	31,9	17,7	0,0	0,1	3,2	0,1	0,0	0,1	0,1
24914		G	-	C	-	-	-	C	C	C	C	-	-	C	C	C	0,0	11,6	24,7	43,9	18,7	0,0	0,1	4,4	0,0	0,0	0,2	0,1
25460	ORF 3a	C	-	-	-	-	-	-	U	-	-	-	-	U	-	-	0,2	0,1	49,6	12,9	26,2	0,0	0,3	0,2	0,3	0,1	0,2	0,1
25515		C	-	-	-	-	-	A	-	A	-	-	-	-	-	-	0,0	0,0	29,4	31,9	37,6	0,1	0,3	0,0	0,0	0,1	0,2	0,2
25517		C	-	-	-	-	-	-	-	-	U	-	-	-	-	-	0,1	0,0	0,0	0,1	9,9	0,0	37,0	0,1	0,0	0,2	0,1	0,1
25521		C	-	-	-	-	-	-	-	-	-	-	-	-	U	-	0,1	0,1	0,0	0,1	3,1	0,1	27,9	0,2	0,1	0,1	0,9	0,0
25563		G	U	-	U	U	U	-	U	-	-	U	U	U	-	-	0,0	46,2	40,2	20,4	27,4	0,0	0,0	5,6	0,1	0,3	0,2	0,1
25710		C	U	-	U	-	U	-	U	-	-	U	U	U	-	-	0,1	42,2	34,4	17,6	26,0	0,0	0,1	7,4	0,1	0,0	0,3	0,0
26204		C	-	-	-	-	-	-	U	-	-	U	-	U	-	-	0,0	23,5	42,9	25,9	28,1	0,0	0,0	3,7	23,0	10,6	0,0	0,0
26681	M	C	-	-	-	-	-	-	-	U	-	-	-	-	-	-	3,1	0,1	0,0	0,1	6,2	49,1	0,3	0,2	0,1	0,1	0,0	0,2
26735		C	U	-	U	-	-	-	-	-	-	U	U	U	U	-	0,0	37,9	43,5	9,1	8,6	0,0	19,6	17,0	0,0	0,1	0,3	0,2
26819		U	-	-	-	-	-	-	G	-	-	-	-	-	-	-	0,1	0,0	0,0	0,0	35,4	0,1	0,0	0,0	0,1	0,0	0,1	0,3
26835		C	-	-	-	-	-	-	U	-	-	-	-	-	-	-	0,0	0,0	0,0	0,1	45,4	0,0	0,0	0,1	0,0	0,0	0,2	0,1
26858		C	-	-	-	U	-	U	-	U	U	-	-	-	-	U	0,0	44,1	48,8	13,5	23,6	0,0	28,6	20,2	0,0	0,0	0,1	0,1
26876		U	C	-	C	-	-	-	C	-	-	C	C	C	C	-	0,0	39,2	46,4	12,6	37,2	0,0	22,5	14,7	0,2	0,2	0,2	0,2
26907		C	-	-	-	-	-	-	U	-	-	U	U	U	-	-	0,0	24,9	48,8	43,7	34,9	0,0	0,0	49,1	21,2	15,8	0,6	0,0
26952		A	-	-	-	-	-	-	-	-	-	-	-	-	G	-	0,0	0,0	0,0	0,0	1,5	0,0	28,3	0,0	0,0	0,0	0,1	0,1
27459	ORF 6 / ORF 7a / ORF 7b / ORF 9b / N	G	-	-	-	-	-	-	-	-	U	-	-	-	-	U	0,0	0,0	0,0	0,1	10,0	0,6	11,1	0,1	0,0	0,0	0,0	2,3
27918		A	-	-	-	-	-	-	-	G	G	-	-	-	G	G	0,3	0,0	0,3	5,1	21,8	0,0	0,0	0,0	0,1	0,0	0,0	0,0
28038		G	-	-	-	-	-	-	-	U	U	-	-	-	U	U	0,0	0,0	1,5	6,4	26,7	0,0	0,0	0,1	0,0	0,1	0,0	0,0
28271		A	-	-	-	U	U	U	U	U	U	U	U	U	U	U	0,1	35,5	0,1	0,0	11,4	0,0	0,1	6,2	0,1	0,3	0,3	0,1
28338		A	-	-	-	-	-	-	-	-	-	-	-	-	-	G	0,1	0,0	0,0	0,0	1,3	0,0	0,0	0,0	0,0	0,0	0,1	0,3
28560		G	-	-	-	U	U	U	U	U	C	U	U	U	U	C	0,1	0,0	0,0	0,1	4,4	0,1	19,2	0,0	0,0	0,0	0,3	0,1
28572		A	-	-	-	-	-	-	-	-	G	-	-	-	-	G	0,1	0,0	0,0	0,1	1,2	0,0	21,0	0,0	0,0	0,0	0,1	0,2
28657		C	-	-	-	-	-	-	-	-	-	-	-	-	-	U	0,2	0,0	0,0	0,0	2,5	0,0	0,6	0,1	0,0	0,0	0,1	0,3
28887		C	-	-	-	-	-	-	-	-	U	-	-	-	-	U	0,2	0,0	0,0	0,0	3,5	0,0	15,5	0,2	0,1	0,1	0,4	0,0
28975		G	C	-	C	-	C	C	C	C	C	C	C	C	C	C	0,4	32,8	0,0	0,0	16,6	0,0	5,5	7,3	0,0	0,1	0,1	0,3
28977		C	-	U	-	U	-	-	-	-	-	-	-	-	-	-	0,0	45,1	2,5	0,0	2,8	0,7	0,0	7,3	0,0	0,0	0,2	0,1
29242		A	-	-	-	U	-	-	-	-	-	-	-	-	-	-	0,1	47,9	0,2	0,1	2,0	0,0	0,1	5,8	0,0	0,2	0,3	0,3
29399		G	A	-	A	A	A	A	A	A	A	A	A	A	A	A	0,0	0,1	0,0	0,0	9,3	0,0	0,0	0,0	0,0	0,1	0,1	0,5
29445		C	-	-	-	-	U	-	U	-	-	-	U	U	-	-	0,0	0,1	31,6	30,4	16,2	0,0	0,0	0,0	0,0	0,2	0,0	0,0
29743		C	-	-	-	-	-	-	-	-	-	-	-	U	-	-	0,1	0,0	0,0	0,0	32,1	0,0	0,0	0,1	0,0	13,5	0,1	0,1

Supplementary Figure S2b.

Nucleotide positions	Nasopharyngeal samples							Culture supernatants				
	September 18th, 2020	May 5th, 2021	June 21st, 2021	July 5th, 2021	August 12th, 2021	October 1st, 2021	November 24th, 2021	May 5th, 2021	June 14th, 2021	August 13th, 2021	November 24th, 2021	December 2nd, 2021
**241**	2150	352	3	1704	20886	164	1666	590	1037	601	134	826
**629**	994	573	121	1725	15751	960	793	1639	1204	1004	1333	2036
**696**	3494	3506	3644	3332	67497	2441	2681	2199	2210	1457	1499	2574
**913**	2521	2860	3041	2330	51997	1753	2001	1205	1600	828	760	1455
**981**	3342	3018	3306	2933	111794	2344	2404	2416	1979	1514	1651	2601
**1192**	837	812	668	1499	59773	1396	736	1963	1374	1402	1612	1952
**1677**	1055	123	236	943	129526	673	646	1717	1372	1169	1202	1635
**1683**	1056	121	236	973	129521	680	681	1690	1368	1171	1172	1635
**1895**	3856	128	229	802	163711	96	155	377	249	209	191	376
**1978**	2898	266	70	316	38991	102	1250	1469	2128	1160	432	1490
**2509**	1618	146	326	508	6193	91	122	259	197	197	178	269
**2594**	663	242	23	391	14494	199	1235	1657	2040	1210	558	2141
**2706**	665	243	20	442	14618	221	1435	1767	1933	1349	609	2476
**3037**	694	395	230	569	53	722	854	1515	938	1119	1345	1603
**3193**	1410	1795	1891	1329	33319	231	582	342	455	189	211	361
**3224**	736	1925	1985	1550	33479	866	1704	980	1360	681	680	967
**3267**	735	2002	1946	1312	33223	1073	1840	1024	1520	562	788	1049
**3340**	734	2127	2112	1698	32456	1510	2003	1122	1705	836	712	1142
**3393**	731	1696	1624	1333	32139	1136	1586	865	1291	602	590	855
**4543**	3340	489	7	1275	304941	247	1546	1516	1733	1069	441	953
**4872**	1522	76	8	143	4024	212	669	2538	1645	1127	1153	3332
**4878**	1521	72	7	118	4012	179	610	2453	1591	1056	1051	3188
**4893**	1520	91	11	127	3991	178	602	2458	1649	1023	1088	3348
**4904**	1520	95	10	117	3986	145	535	2329	1682	926	976	3274
**5278**	850	828	92	343	718	67	151	169	241	78	71	192
**5629**	1774	799	189	1765	51104	164	617	389	472	212	42	388
**5648**	1181	797	189	1850	51155	486	1860	1017	1553	703	374	1190
**5986**	571	149	224	199	913	383	528	1207	849	940	1075	1349
**6629**	1811	410	284	699	162979	893	857	1394	747	961	1139	1593
**6633**	1811	425	302	733	163229	937	902	1457	816	1005	1204	1635
**7113**	1974	30	0	95	288760	6	194	415	297	313	170	473
**7185**	1653	8	2	68	284759	15	578	1427	810	980	592	1433
**7279**	1653	15	6	110	285215	18	770	1630	1162	1312	860	1701
**8191**	2290	1560	928	1493	177777	1106	1893	1083	1614	807	824	1146
**8770**	1971	108	2	454	153168	28	1543	1035	1537	900	325	1076
**8950**	2911	1251	1238	2031	259162	1318	1912	2233	1550	1597	1349	2443
**9042**	963	840	955	1326	107695	1244	778	1495	884	956	1258	1906
**9120**	961	1099	1287	1677	106579	1590	983	1786	1287	1187	1539	2316
**9501**	1692	87	0	322	4013	23	516	358	688	266	52	267
**9526**	1724	186	1	491	4020	57	2429	1801	1370	591	131	1792
**9626**	1488	2	0	4	181	4	162	340	1708	319	28	660
**9783**	1493	6	0	2	1111	1	155	261	1631	236	23	428
**9792**	2650	6	1	2	22549	1	45	81	504	57	5	104
**9811**	2735	395	178	151	24543	173	718	812	1867	589	261	987
**9972**	1264	682	223	251	24799	357	1396	870	2043	904	594	1175
**11083**	1784	3073	3301	2742	114282	2212	2593	1758	2729	1391	1087	1925
**11208**	1800	2632	2754	2418	114516	1884	2563	1484	2366	1089	925	1684
**11365**	3333	2531	2218	2663	167240	1993	3056	3704	3270	2401	2028	3693
**11497**	1557	285	7	695	54023	509	910	2039	1649	1253	1119	2203
**12459**	2225	1415	896	2354	63912	1377	2333	3008	1870	1939	1796	3089
**12592**	916	438	688	1380	23200	981	877	1734	1183	1405	1599	1755

Supplementary Figure S2b (continued)

Nucleotide positions	Nasopharyngeal samples							Culture supernatants				
	September 18th, 2020	May 5th, 2021	June 21st, 2021	July 5th, 2021	August 12th, 2021	October 1st, 2021	November 24th, 2021	May 5th, 2021	June 14th, 2021	August 13th, 2021	November 24th, 2021	December 2nd, 2021
**13506**	751	19	8	79	7784	6	763	2084	2239	1345	433	2188
**13517**	751	26	8	98	7798	6	857	2324	2535	1517	479	2448
**13860**	2406	693	1098	1297	20082	869	913	2767	1799	1537	1948	2481
**13936**	5135	969	1413	1387	160834	385	430	920	601	570	626	886
**13993**	2826	1931	1232	1485	143197	1240	2017	2074	1456	743	510	1177
**14408**	320	876	155	1628	21272	1343	844	1654	1575	1176	1243	1774
**15766**	1689	1008	1839	1791	47916	1480	840	1480	1395	1162	1463	1717
**16128**	3156	3403	2881	2744	57062	687	703	501	650	342	153	554
**16675**	811	818	448	455	31287	727	1562	901	1055	423	386	1129
**16751**	1830	978	569	546	31093	276	525	297	429	149	154	369
**16889**	1236	209	12	292	233	509	1206	2272	1850	1715	1421	2741
**17019**	1237	137	19	175	225	324	791	1510	1304	1017	849	1838
**17122**	3716	2711	1588	2183	68134	1562	3401	2728	2586	1595	1649	3410
**17125**	3716	2527	1485	2054	68137	1435	3221	2496	2440	1420	1535	3126
**17251**	2512	2303	1468	2283	69806	1686	2221	1252	1783	992	1046	1468
**17721**	2530	1170	552	2074	31757	862	2288	2056	1719	1380	1121	1972
**17738**	2522	947	408	1750	31777	598	1874	1541	1415	1068	697	1447
**18023**	2671	1143	1496	2294	109388	1253	2310	2414	1877	1614	1632	2330
**18877**	1328	1598	2688	2248	62018	2114	1310	1911	1848	1464	1872	2269
**19186**	1445	1670	1687	1462	37080	1031	1504	926	1445	656	604	956
**19547**	14	97	78	40	81	124	251	628	396	508	482	861
**19947**	148	71	19	224	392	58	519	1174	870	1040	648	1478
**20930**	409	2758	2087	2111	7742	1685	1855	1222	2118	989	831	1493
**21219**	157	23	127	26	8	93	396	692	1130	873	529	1483
**21232**	157	25	132	29	7	104	400	690	1091	884	530	1457
**21575**	221	363	132	132	3101	294	580	271	914	518	288	817
**21765**	2025	756	1110	1045	3515	702	571	1409	1242	624	800	1091
**21766**	2025	717	1076	925	3513	552	445	1426	1272	494	630	869
**21767**	2024	723	1090	934	3513	543	440	1461	1304	488	621	861
**21768**	2024	721	1075	929	3513	543	440	1454	1267	488	621	861
**21769**	2024	711	1045	915	3513	544	441	1414	1223	489	623	863
**21770**	2024	710	1035	933	3513	575	466	1370	1165	526	677	919

Supplementary Figure S2b (continued)

Nucleotide positions	Nasopharyngeal samples							Culture supernatants				
	September 18th, 2020	May 5th, 2021	June 21st, 2021	July 5th, 2021	August 12th, 2021	October 1st, 2021	November 24th, 2021	May 5th, 2021	June 14th, 2021	August 13th, 2021	November 24th, 2021	December 2nd, 2021
**21991**	3898	1347	1126	1191	11092	80	70	1573	96	80	106	144
**21992**	3900	1346	1121	1185	11090	80	71	1578	96	80	106	145
**21993**	3900	1338	1117	1176	11088	82	74	1583	104	81	107	151
**22992**	1241	9	0	175	151073	12	186	980	1363	997	95	673
**23063**	1226	16	3	214	150290	7	243	1142	1610	1029	116	776
**23188**	3006	2370	2725	2115	165844	1184	1572	1338	1947	1026	481	1333
**23271**	1840	2849	3112	2196	19307	1490	1827	1063	2417	706	601	1437
**23277**	1840	2957	3275	2345	19312	1631	1980	1151	2603	831	681	1571
**23403**	1837	2753	3033	2309	20084	1749	1974	1168	2358	921	714	1643
**23415**	1834	2889	3168	2442	20072	1869	2098	1254	2544	986	767	1754
**23525**	782	726	1039	1136	1106	956	860	1377	1761	1102	1060	1929
**23598**	784	787	1096	1200	689	988	977	202	1647	1185	1185	2070
**23604**	784	828	1135	1228	689	995	976	107	1702	1192	1162	2071
**23709**	783	703	957	996	688	778	868	1384	1387	862	921	1605
**23948**	2775	352	243	434	51870	216	998	660	955	397	221	529
**24023**	2776	469	308	478	51684	280	1282	724	1216	566	304	722
**24506**	1061	1248	336	993	40365	660	1497	793	1485	562	566	1000
**24914**	953	1286	2252	2139	56056	1706	1017	1808	1896	1247	1577	2169
**25460**	942	787	1402	1411	109844	1314	794	2225	1764	1233	1424	1744
**25515**	942	774	1492	1357	109838	1229	910	2476	1803	1411	1331	1895
**25517**	942	744	1477	1346	109841	1159	903	2407	1680	1324	1281	1812
**25521**	942	753	1386	1246	109845	1121	904	2390	1679	1313	1262	1783
**25563**	942	604	1079	1096	109851	1004	684	2107	1299	899	1122	1489
**25710**	1647	351	131	493	58375	369	1455	2327	1623	826	565	1447
**26204**	1014	221	35	239	22478	87	215	652	453	179	391	633
**26681**	977	1114	950	1537	1767	1408	1001	2401	2245	1384	1753	3572
**26735**	979	1201	1084	1729	1804	1681	1125	2807	2065	1327	1686	4015
**26819**	979	1224	1103	1428	5035	1482	1097	2895	2426	1402	1725	4763
**26835**	991	1301	1184	1407	5549	1238	1063	2756	2116	1243	1538	4249
**26858**	2548	1491	1290	1574	32691	308	405	1066	555	395	459	1141
**26876**	2553	1214	1042	1322	32927	1149	1834	4224	2713	1901	1484	3997
**26907**	2347	233	43	247	32445	10	299	864	631	336	120	707
**26952**	1594	222	45	282	31569	33	911	3053	2445	1381	409	3308
**27459**	1380	1048	1563	1661	114777	361	262	761	498	344	431	820
**27918**	383	618	630	254	133	14	8	1189	1151	771	14	20
**28038**	383	774	915	362	135	27	22	1690	1456	1007	41	56
**28271**	1896	2572	3143	2135	92933	1762	1829	1473	1720	760	647	1217
**28338**	1894	3302	3787	2615	92839	2051	2050	1563	2017	925	876	1423
**28560**	179	829	1450	1667	12470	1260	798	2017	1535	1200	1277	2103
**28572**	179	907	1533	1789	12474	1368	1003	2162	1653	1295	1390	2429
**28657**	179	1106	1816	2053	12462	1579	1057	2834	2155	1644	1663	3113
**28887**	1241	827	66	212	7521	222	956	2372	2567	1083	555	2506
**28975**	1241	808	41	139	7558	151	888	2328	1816	754	382	2595
**28977**	1241	548	40	127	7569	141	841	2227	1672	704	354	2478
**29242**	1366	771	461	1371	48806	766	838	2431	1909	1475	1265	2651
**29399**	359	1382	172	329	5301	593	853	1056	1414	687	501	1073
**29445**	360	1429	133	339	5326	494	749	927	1166	548	425	908
**29743**	385	450	128	298	4362	483	624	1312	940	1035	765	1412

Supplementary Figure S3. Detailed content of amplicons corresponding to positions 3,100-4,570 of the SARS-CoV-2 genome and retrieved from respiratory samples collected on May 5th, 2021. Amplicons were first sorted in order to keep continuous reads for analysis. The number and percentage of each type of reads are indicated. B.1.160 variant regions are indicated by a blue color; Alpha variant regions are indicated by a green color; patient-specific mutations are indicated by a red color. Reads were visualized using the IGV software [3].

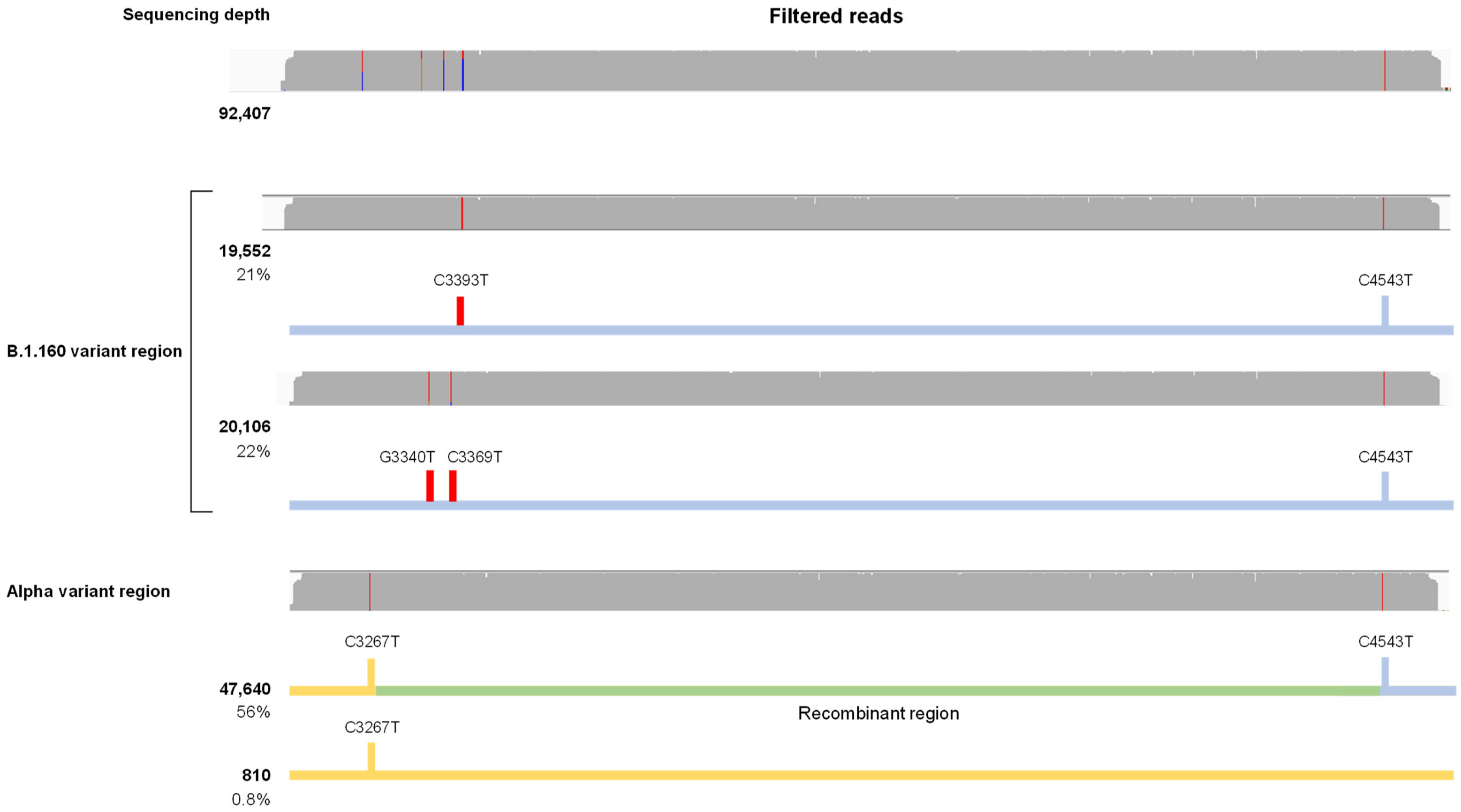

Supplementary Figure S4. Detailed content of amplicons corresponding to positions 3,100-4,570 of the SARS-CoV-2 genome and retrieved from respiratory samples collected on August 12th, 2021. Amplicons were first sorted in order to keep continuous reads for analysis. The number and percentage of each type of reads are indicated. B.1.160 variant regions are indicated by a blue color; Alpha variant regions are indicated by a green color; patient-specific mutations are indicated by a red color. Reads were visualized using the IGV software [3].

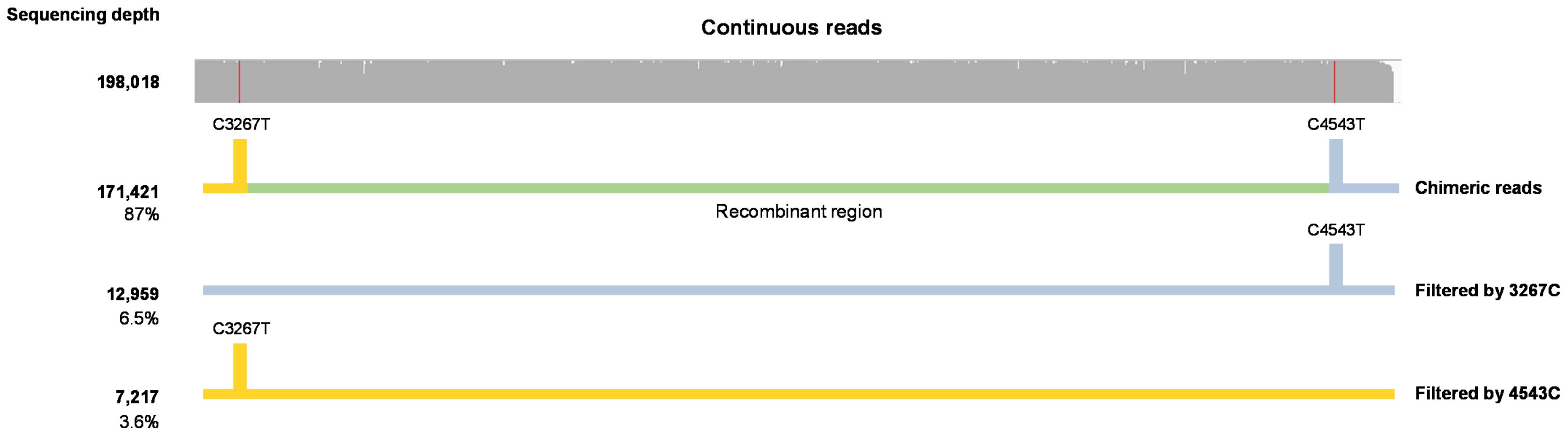

Supplementary Figure S5. Detailed content of amplicons corresponding to positions 24,880-29,010 of the SARS-CoV-2 genome and retrieved from respiratory samples collected on August 12th, 2021. Amplicons were first sorted in order to keep continuous reads for analysis. The number and percentage of each type of reads are indicated. B.1.160 variant regions are indicated by a blue color; Alpha variant regions are indicated by a green color; patient-specific mutations are indicated by a red color. Reads were visualized using the IGV software [3].

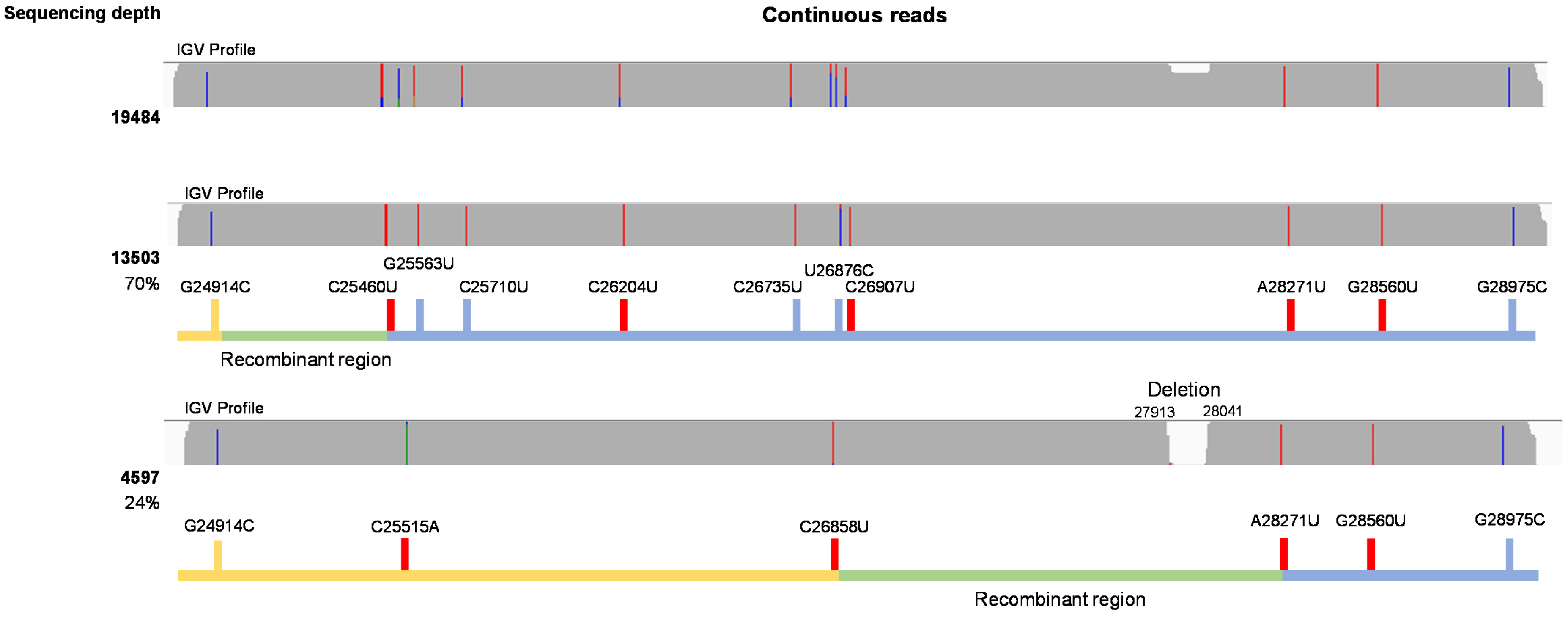

4. SUPPLEMENTARY TABLES

Supplementary Table S1. List of GISAID identifiers for sequences used in the present study.

All genomes were obtained in our laboratory (University hospital institute Méditerranée Infection, Marseille, France). The GISAID sequence database is accessible at: (https://www.gisaid.org/) [4].

Source sample		GISAID identifier
Respiratory samples		
	Case-patient's sequences	EPI_ISL_10816731
		EPI_ISL_10816733
		EPI_ISL_10816742
		EPI_ISL_10816743
		EPI_ISL_10816744
		EPI_ISL_11030507
		EPI_ISL_6332079
	Most similar sequences from our database	EPI_ISL_10816729
		EPI_ISL_10816740
		EPI_ISL_2757096
		EPI_ISL_3054898
		EPI_ISL_3054899
		EPI_ISL_3055037
		EPI_ISL_3055223
		EPI_ISL_3055225
		EPI_ISL_3055227
		EPI_ISL_3055234
		EPI_ISL_3055235
		EPI_ISL_3830105
		EPI_ISL_3830215
		EPI_ISL_3831874
		EPI_ISL_3933559
		EPI_ISL_3933565
		EPI_ISL_3933569
		EPI_ISL_3933571
		EPI_ISL_3933572
		EPI_ISL_3934656
		EPI_ISL_3934682
		EPI_ISL_3934683
		EPI_ISL_4394365
		EPI_ISL_4944362
		EPI_ISL_4944364
		EPI_ISL_4951784
		EPI_ISL_4951785
		EPI_ISL_6737742
		EPI_ISL_7376142
		EPI_ISL_7376355

Supplementary Table S1 - continued

		EPI_ISL_8321213
		EPI_ISL_8321223
		EPI_ISL_8321228
		EPI_ISL_8321229
		EPI_ISL_8321241
		EPI_ISL_8321242
		EPI_ISL_8321256
		EPI_ISL_8709907
		EPI_ISL_8709910
		EPI_ISL_8711731
		EPI_ISL_9773781
		EPI_ISL_9774389
		EPI_ISL_9774894
		EPI_ISL_9774895
Culture supernatants		
	Case-patient's sequences	EPI_ISL_10816730
		EPI_ISL_10816732
		EPI_ISL_10816734
		EPI_ISL_10816735
		EPI_ISL_10816738

Supplementary Table S2. Sampling time and location for sequences used in the present study.

GISAID identifier	Date	Location
EPI_ISL_3830215	2021-03	Europe / France / Provence-Alpes-Cote d'Azur /Marseille
EPI_ISL_10816729	2020-11	Europe / France / Provence-Alpes-Cote d'Azur / Marseille
EPI_ISL_10816740	2021-03	Europe / France / Provence-Alpes-Cote d'Azur / Marseille
EPI_ISL_10816741	2021-11	Europe / France / Provence-Alpes-Cote d'Azur / Marseille
EPI_ISL_2757096	2020-11-07	Europe / France / Provence-Alpes-Cote d'Azur / Marseille
EPI_ISL_3054898	2020-09-11	Europe / France / Provence-Alpes-Cote d'Azur / Marseille
EPI_ISL_3054899	2020-09-14	Europe / France / Provence-Alpes-Cote d'Azur / Marseille
EPI_ISL_3055037	2020-09-12	Europe / France / Provence-Alpes-Cote d'Azur / Marseille
EPI_ISL_3055223	2020-09-15	Europe / France / Provence-Alpes-Cote d'Azur / Marseille
EPI_ISL_3055225	2020-09-15	Europe / France / Provence-Alpes-Cote d'Azur / Marseille
EPI_ISL_3055227	2020-09-15	Europe / France / Provence-Alpes-Cote d'Azur / Marseille
EPI_ISL_3055234	2020-09-17	Europe / France / Provence-Alpes-Cote d'Azur / Marseille
EPI_ISL_3055235	2020-09-17	Europe / France / Provence-Alpes-Cote d'Azur / Marseille
EPI_ISL_3830105	2021-03	Europe / France / Provence-Alpes-Cote d'Azur / Marseille
EPI_ISL_3831874	2020-08	Europe / France / Provence-Alpes-Cote d'Azur / Marseille
EPI_ISL_3933559	2020-11	Europe / France / Provence-Alpes-Cote d'Azur / Marseille
EPI_ISL_3933565	2020-09	Europe / France / Provence-Alpes-Cote d'Azur / Marseille
EPI_ISL_3933569	2020-08	Europe / France / Provence-Alpes-Cote d'Azur / Marseille
EPI_ISL_3933571	2020-08	Europe / France / Provence-Alpes-Cote d'Azur / Marseille
EPI_ISL_3933572	2020-08	Europe / France / Provence-Alpes-Cote d'Azur / Marseille
EPI_ISL_3934656	2021-04	Europe / France / Provence-Alpes-Cote d'Azur / Marseille
EPI_ISL_3934682	2021-04	Europe / France / Provence-Alpes-Cote d'Azur / Marseille
EPI_ISL_3934683	2021-03	Europe / France / Provence-Alpes-Cote d'Azur / Marseille
EPI_ISL_4394365	2021-04	Europe / France / Provence-Alpes-Cote d'Azur / Marseille
EPI_ISL_4944362	2021-07	Europe / France / Provence-Alpes-Cote d'Azur / Marseille
EPI_ISL_4944364	2021-07	Europe / France / Provence-Alpes-Cote d'Azur / Marseille
EPI_ISL_4951784	2021-04	Europe / France / Provence-Alpes-Cote d'Azur / Marseille
EPI_ISL_4951785	2021-04	Europe / France / Provence-Alpes-Cote d'Azur / Marseille
EPI_ISL_6737742	2021-01	Europe / France / Provence-Alpes-Cote d'Azur / Marseille
EPI_ISL_7376142	2021-01	Europe / France / Provence-Alpes-Cote d'Azur / Marseille
EPI_ISL_7376355	2021-01	Europe / France / Provence-Alpes-Cote d'Azur / Marseille
EPI_ISL_8321213	2020-10	Europe / France / Provence-Alpes-Cote d'Azur / Marseille
EPI_ISL_8321223	2020-10	Europe / France / Provence-Alpes-Cote d'Azur / Marseille
EPI_ISL_8321228	2020-10	Europe / France / Provence-Alpes-Cote d'Azur / Marseille
EPI_ISL_8321229	2020-10	Europe / France / Provence-Alpes-Cote d'Azur / Marseille
EPI_ISL_8321241	2020-10	Europe / France / Provence-Alpes-Cote d'Azur / Marseille
EPI_ISL_8321242	2020-10	Europe / France / Provence-Alpes-Cote d'Azur / Marseille
EPI_ISL_8321256	2020-10	Europe / France / Provence-Alpes-Cote d'Azur / Marseille
EPI_ISL_8709907	2021-11	Europe / France / Provence-Alpes-Cote d'Azur / Marseille
EPI_ISL_8709910	2022-01	Europe / France / Provence-Alpes-Cote d'Azur / Marseille
EPI_ISL_8711731	2021-03	Europe / France / Provence-Alpes-Cote d'Azur / Marseille
EPI_ISL_9773781	2020-03	Europe / France / Provence-Alpes-Cote d'Azur / Marseille
EPI_ISL_9774389	2020-03	Europe / France / Provence-Alpes-Cote d'Azur / Marseille
EPI_ISL_9774894	2021-04	Europe / France / Provence-Alpes-Cote d'Azur / Marseille
EPI_ISL_9774895	2021-04	Europe / France / Provence-Alpes-Cote d'Azur / Marseille

*Supplementary Table S3. Identifiers of r*aw data deposited in the European Bioinformatics Institute (EMBL-EBI) sequence database (https://www.ebi.ac.uk/)

a.

Raw data EMBL-EBI identifier	GISAID identifier	Sampling date	Time from diagnosis (days)	Next-generation sequencing technology, instrument
ERR9671577	EPI_ISL_6332079	September 18th, 2020	0	Illumina, NovaSeq
ERR9671578	EPI_ISL_10816743	May 5th, 2021	229	Illumina, NovaSeq
ERR9671579	EPI_ISL_11030507	June 21st, 2021	276	Illumina, NovaSeq
ERR9671580	EPI_ISL_10816731	July 5th, 2021	290	Illumina, NovaSeq
ERR9692770	EPI_ISL_10816742	August 12th, 2021	328	Nanopore, GridION
ERR9671581	EPI_ISL_10816744	October 1st, 2021	378	Illumina, NovaSeq
ERR9671582	EPI_ISL_10816733	November 24th, 2021	432	Illumina, NovaSeq

b.

Raw data EMBL-EBI identifier	GISAID identifier	Sampling date of the nasopharyngeal sample	Time to cytopathic effect (days)	Next-generation sequencing technology, instrument
ERR9671585	EPI_ISL_10816730	May 5th, 2021	8	Illumina, NovaSeq
ERR9671583	EPI_ISL_10816732	June 14th, 2021	4	Illumina, NovaSeq
ERR9671584	EPI_ISL_10816734	August 13th, 2021	4	Illumina, NovaSeq
ERR9671586	EPI_ISL_10816735	November 24th, 2021	5	Illumina, NovaSeq
ERR9671587	EPI_ISL_10816738	December 2nd, 2021	7	Illumina, NovaSeq

c.

Raw data EMBL-EBI identifier	GISAID identifier / pre-sequencing approach	Sequenced region	Sampling date
ERR9683059	EPI_ISL_10816742 / RT-Klenow	Whole genome	August 12th, 2021
ERR9683060	EPI_ISL_10816743 / H2	3,100-4,570	May 5th, 2021
ERR9683061	EPI_ISL_10816742 / H3	24,813-29,074	August 12th, 2021
ERR9738766	EPI_ISL_10816742 / H2	3,100-4,570	August 12th, 2021

References

1. La Scola, B.; Le Bideau, M.; Andreani, J.; et al. Viral RNA load as determined by cell culture as a management tool for discharge of SARS-CoV-2 patients from infectious disease wards. *Eur J Clin Microbiol Infect Dis.* **2020**, *39*, 1059-1061.
2. Colson, P.; Fournier, PE.; Chaudet, H.; et al. Analysis of SARS-CoV-2 Variants From 24,181 Patients Exemplifies the Role of Globalization and Zoonosis in Pandemics. *Front Microbiol.* **2022**, *12*, 786233. doi: 10.3389/fmicb.2021.786233.
3. Robinson, J.T.; Thorvaldsdottir, H.; Wenger, A.M.; Zehir, A.; Mesirov, J.P. Variant Review with the Integrative Genomics Viewer (IGV). *Cancer Research* **2017**, *77*, 31-34.
4. Alm, E.; Broberg, EK.; Connor, T.; et al. Geographical and temporal distribution of SARS-CoV-2 clades in the WHO European Region, January to June 2020. *Euro Surveill.* **2020**, *25*, 2001410.
